# Microglia-containing neural organoids as brain microphysiological systems for long-term culture

**DOI:** 10.3389/fncel.2025.1616470

**Published:** 2025-10-02

**Authors:** Alex Rittenhouse, Caroline Krall, Jesse Plotkin, Dowlette-Mary Alam El Din, Breanne Kincaid, Jason Laird, Lena Smirnova

**Affiliations:** 1 Center for Alternatives to Animal Testing, Department of Environmental Health and Engineering, Bloomberg School of Public Health, Johns Hopkins University, Baltimore, MD, United States; 2 Department of Molecular and Comparative Pathobiology, Johns Hopkins University School of Medicine, Baltimore, MD, United States

**Keywords:** microphysiological systems (MPS), neural organoids, microglia, iPSC-derived 3D models, new approach methodologies (NAMs)

## Abstract

Microglia, essential for brain development, homeostasis, and neuroinflammation, originate from the yolk sac during embryogenesis and migrate into the developing brain. Because of this developmental origin, many brain organoid models naturally lack microglia and require co-culture. To address this issue, we developed a microglia-integrated brain organoid model (immune-competent brain microphysiological system, *μ*bMPS) by aggregating hiPSC-derived neural and microglia progenitors in U-bottom 96-well plates, allowing controlled and reproducible incorporation of microglia progenitors. We demonstrated that microglia integrated, matured, and survived long-term in the neural environment without the need for costly exogenous microglia-specific growth factors or cytokines. We maintained microglia-containing organoids for over 9 weeks, demonstrating functional activity, phagocytosis, and neuroinflammatory responses. The μbMPS also exhibited enhanced neuronal activity and maturity, providing a scalable, reproducible model for neurodevelopment, disease modeling, and neurotoxicology research.

## Introduction

Microglia are brain-resident immune cells that are essential for maintaining brain function and homeostasis during both development and adulthood. They originate from the yolk sac in early embryogenesis and migrate into the developing brain, appearing as early as gestational week 4 (Monier et al., 2007), where they engage in extensive bidirectional interactions with developing neurons. Microglial differentiation and maintenance are supported by neuron-derived cytokines, including colony-stimulating factor 1 (CSF-1), interleukin 34 (IL-34), and transforming growth factor-beta (TGF-*β*). In turn, microglia influence neuronal development by secreting factors such as tumor necrosis factor (TNF) and nerve growth factor (NGF) (Matejuk and Ransohoff, 2020).

During later stages of brain development, microglia play a crucial role in synaptic pruning, primarily by identifying weak or abnormal synapses through complement molecules (C1q and C3) or fractalkine signaling. While astrocytes can also contribute to this process, microglia are generally considered the primary mediators of targeted synaptic pruning (Mordelt and de Witte, 2023). In the mature brain, microglia continue to engage in bidirectional communication with neurons and other glial cells during both homeostasis and conditions of disease or injury. For instance, microglia migrate toward extracellular ATP released by overactive neurons to help regulate neuronal activity. This interaction may also stimulate the release of brain-derived neurotrophic factor (BDNF) from microglia, thereby influencing neuronal plasticity and function (Matejuk and Ransohoff, 2020).

Microglia are also crucial to excitatory signaling and network regulation, working in conjunction with astrocytes and glutamatergic pre- and post-synaptic terminals to form the “quad-partite synapse.” They modulate synapses through direct contact and secreted factors, playing region-specific roles while contributing to neuronal network modulation across the brain (Schafer et al., 2013). Beyond their neurodevelopmental functions, microglia serve as the brain’s resident immune cells, responsible for clearing dead cells, pathogens, and pathological aggregates, as well as responding to inflammatory signals from other cells (Matejuk and Ransohoff, 2020; Luo and Wang, 2024). Furthermore, mutations affecting microglia and changes in their morphologies have been implicated and associated with many adult CNS diseases, including, but not limited to, Alzheimer’s disease (Wickstead, 2023), Huntington’s disease (Palpagama et al., 2019), Parkinson’s disease (Trainor et al., 2024), and multiple sclerosis (Fagiani et al., 2024).

Despite the crucial role of microglia, many human-induced pluripotent stem cell (hiPSC)-derived brain organoid models lack these cells. This is because, unlike neurons, astrocytes, and oligodendrocytes—which originate from the neuroectoderm—microglia arise from the yolk sac during primitive hematopoiesis (Ginhoux et al., 2013). As a result, organoids generated solely from neuroectodermal cells inherently lack microglia. Several approaches have been developed to incorporate microglia into brain organoids, each with specific strengths and limitations (Fagiani et al., 2024; Wenzel et al., 2024; Ormel et al., 2018; Bodnar et al., 2021; Popova et al., 2021; Park et al., 2023; Sabate-Soler et al., 2022; Schafer et al., 2023; Buonfiglioli et al., 2025; Fagerlund et al., 2021; Cakir et al., 2022; Kalpana et al., 2025; Wu et al., 2024; Farahani et al., 2023; Muffat et al., 2016; Xu et al., 2021; Song et al., 2019; Abreu et al., 2018) (summarized and compared in Table 1; Supplementary Figure S1). Among these, co-culturing microglia with neural progenitors provides the most reproducible approach for forming 3D cultures. However, published protocols typically require microglia-specific factors or media, or they fail to maintain microglia throughout the entire culture period.

**Table 1:** Literature review, summarizing existing microglia-containing organoid models.

Authors	bMPS description	Microglia source	Integration method	Microglia present from	Media altered
Farahani et al. (2023)	Neural Assembloids	iPSC	Mature organoids (> day 50) were individually pipetted one per well of a 96-well plate, then microglia were added and allowed to aggregate	>7-weeks	Yes, stemcell technologies supplement 2
Muffat et al. (2016)	Neural Co-Culture	iPSC	Microglia were added to 4-week-old neuronal cultures in a transwell system or combined in 96-well ULA plates at 1:10 ratio with microglia-like cells	4 Weeks	Yes, IL-34 and CSF1
Abreu et al. (2018)	Human BrainSpheres	Immortalized microglia	Microglia were added to 7-week-old spheres in suspension in a 6-well plate and allowed to invade for over 24 h, then all assembloids were moved to a new plate	7-weeks	No
Fagiani et al. (2024)	Glia-enriched organoids	iPSC	Microglia added to 8-week-old organoids, 200,000 cells/well of a 6-well plate	8-Weeks	No
Fagerlund et al. (2021)	Cerebral Organoids	iPSC	Erythromyeloid progenitors incorporated into already formed cerebral organoids at day 30	D30 (~4 W)	No
Wu et al. (2024)	Cortical Organoids	iPSC	Mature (Day 120) organoids were individually placed into a ULA 96-well plate, then microglia were added on top with a 50/50 microglia/cortical media mixture	Day 120 (~17 W)	Yes, IL-34, TGF beta1, M-CSF
Sabate-Soler et al. (2022)	Midbrain organoids	iPSC	Microglia were added to the formed midbrain organoids on day 15 of dopaminergic differentiation	Day 15 (2 W)	Yes, GM-CSF and IL-34
Buonfiglioli et al. (2025)	Cerebral Organoids	iPSC	Hematopoietic precursor cells were added to formed Cerebral Organoids between days 25 and 35	Day 25–35 (~4 W)	Yes, IL-34, TGF-beta, M-CSF
Park et al. (2023)	Cerebral Organoids	iPSC	Microglia were added to already formed embryoid body-derived cerebral organoids (day 26)	Day 26 (~4 W)	Yes, CSF1
Song et al. (2019)	Dorsal/Ventral Spheroids	iPSC	Organoids and microglia were combined at a 4:1 ratio on day 33 of organoid differentiation	Day 33 (~5 W)	No
Schafer et al. (2023)	Forebrain organoid	iPSC	Erythroid myeloid progenitors added to formed bMPS on day 42	Day 42 (6 W)	Yes, CSF-1, TGFb, and IL-34
Wenzel et al. (2024)	Brain organoids	iPSC	Compared innately developing microglia containing organoids to organoids containing iPSC-derived microglia integrated at day 60	EB innate	No
Bodnar et al. (2021)	Cerebral Organoids	iPSC	Innately present microglia in embryoid body-derived cerebral organoids	First observed ~2 W	Lower heparin
Ormel et al. (2018)	Cerebral Organoids	iPSC	Innately present microglia in embryoid body-derived cerebral organoids	First observed ~3.5 W	Lower heparin
Cakir et al. (2022)	Cortical organoids	iPSC	CRISPRed cells to integrate the doxycycline-inducible PU.1 promoter, doxy drives microglia fate. Incorporated 1:10 CRISPR cells, normal cells at aggregation, and microglia-like cells present from day 30	First observed ~4 W	Doxycycline induction
Xu et al. (2021)	Cerebral Organoids	iPSC	Microglia and NPCs were combined in 96-well plates at a 7:3 ratio, then pooled into 6-well plates	From formation	Yes, M-CSF, IL-3, IL-34, GM-CSF
Kalpana et al. (2025)	Cortical organoids	iPSC	Describe two methods: (1) involves adding microglia to already formed embryoid bodies between days 15–19. (2) involves mixing microglia and NPCs from monoculture in 96-well plates	Method 1: Day 15, Method 2: From formation	Both methods included added IL-34 and GM-CSF
Popova et al. (2021)	Brain organoids	fetal human microglia, iPSC	Fetal microglia were added to week 5 or week 15 organoids	Week 5 or 15	No

In this study, we aimed to develop a novel, highly standardized method for generating neural organoids with integrated hiPSC-derived microglia that persist throughout the whole lifespan of the organoid without the need for microglia-specific media. This design allows us to study microglial roles from early development through mature homeostasis, including synaptic pruning and immune profiling. By utilizing hiPSCs rather than primary cells, our approach enhances human relevance and can be adapted for disease modeling with patient-derived stem cells. Additionally, the protocol prioritizes high reproducibility within and between batches while avoiding the need for exogenous growth factors, providing a robust and scalable tool for both developmental research and studies of responses to environmental exposures.

## Materials and methods

### Cell culture (hiPSC, microglia, NPC, microglia integration, brain microphysiologic system)

A commercially available female hiPSC line (NIBSC-8, UK Stem Cell Bank) was maintained at 37 °C, with 5% CO2 and 5% O2 according to the manufacturer’s recommendations in mTESR Plus medium (STEMdiff™, Catalog #100-0276). It was >90% triple positive for standard iPSC markers (OCT4, SOX2, SSEA4), as evaluated with immunofluorescence. All cell batches were confirmed to be mycoplasma-free and contained no chromosomal aberrations, as evaluated with karyotyping (ThermoFisher Scientific Karyostat+). The cell line was authenticated with short tandem repeat profiling (Johns Hopkins Genomic Resources Core Facility).

Microglial differentiation was performed in three phases. First, hiPSCs (passage 13–17) were differentiated into hematopoietic progenitor cells (HPCs) utilizing the STEMdiff™ Hematopoietic Kit (STEMCELL Technologies™, Catalog #05310), according to the manufacturer’s recommendations. Subsequently, HPCs were differentiated into premature microglia (PMs) with the STEMdiff™ Microglia Differentiation Kit (STEMCELL Technologies™, Catalog #100-0019), with terminal maturation, using the STEMdiff™ Microglia Maturation Kits (STEMCELL Technologies™, Catalog #100-0020). This protocol uses a single medium to differentiate HPCs into PMs over 24 days, with the addition of a final supplement to initiate maturation over an additional 4–12 days. For all integration experiments, premature microglia (PM, day 24 of microglia differentiation) were used, whereas for monoculture experiments, day 24 premature microglia were matured for 8 days prior to assays.

Neural progenitor cells (NPCs) were differentiated as previously described (Romero et al., 2023). Briefly, hiPSCs (passages 7–15) were maintained in mTESR Plus medium on Vitronectin (ThermoFisher Scientific, Catalog# A14700)-coated plates (ThermoFisher Scientific, Catalog #08-772-49). They were then plated at low density on Matrigel (Corning, Catalog# 354277)-coated plates (ThermoFisher Scientific, Catalog #140675) and differentiated into NPCs using Gibco neural induction medium (ThermoFisher Scientific, Catalog #A1647801) for 7 days, following the manufacturer’s instructions. NPCs were then cultured in Neural Expansion Medium, made of Neurobasal/ADVANCED DMEM/F12 media and induction supplement (ThermoFisher Scientific, Catalog # A1647801), as previously described (Romero et al., 2023), for at least five passages with stable and homogeneous morphology prior to all experiments. NPC were differentiated at 37 °C, with 5% CO2 and 5% O2 and then moved to a 20% O2, 5% CO2 incubator after passage 5. Banked NPCs were over 90% double positive for NPC markers (SOX2, Nestin), as confirmed by immunofluorescence (Romero et al., 2023).

To produce a standard brain MPS, we followed our previously published protocol (Romero et al., 2023) and refer to it here as bMPS^6-6^, indicating that the MPS was generated and grown in 6-well plates. Briefly, bMPS^6-6^ were established by plating 2×10^6^ NPCs (no higher than passage 15) in non-coated 6-well plates (Corning, Catalog # 351146), then maintained under constant gyratory shaking (88 rpm and 19 mm orbit) to enable sphere formation. After 2 days, the Neural Expansion Medium was changed to Differentiation Medium (Neurobasal^®^ Plus Medium (ThermoFisher Scientific, Catalog # A3653401) supplemented with 1x B-27 Plus (ThermoFisher Scientific, Catalog # A3582801), 1% GlutaMAX (ThermoFisher Scientific, Catalog #35050061), 0.01 μg/mL human recombinant GDNF (GeminiBio, Catalog# 300-121P-010), 0.01 μg/mL human recombinant BDNF (GeminiBio, Catalog #300-104P-1MG)), and 1% pen/strep/glutamine (ThermoFisher Scientific, Catalog#10378016) to support cellular differentiation and maturation. Cultures were maintained at 37◦C, 5% CO2 and 20% O2 for up to 9 weeks, with two-thirds of the medium exchanged every 2 days.

We assayed three different methods for generating microglia-containing MPS (μbMPS) (Supplementary Figure S2). Integration technique 1: bMPS^6-6^ was generated as previously described (2×10^6^ single-cell suspension of NPC added to a 6-well plate with gyratory shaking), then grown for 3 days. Subsequently, PMs were added (3×10^5^/well) to the wells. For the first 24 h post-integration, plates were incubated statically at 37 °C with 5% CO_2_ with brief shaking every 6 h by an automatic timer to facilitate attachment of the PMs to the bMPS without organoid aggregation. After 24 h, cultures were returned to constant gyratory shaking (88 rpm, 19 mm orbit) for 4 weeks. Integration technique 2: 3×10^5^ PMs and 2×10^6^ NPCs were added to each well of a non-coated 6-well plate. Cultures were maintained with constant gyratory shaking at 88 rpm and a 19 mm orbit for 4 weeks. Integration technique 3 was modified from Xu et al. (2021); 3,000 PMs and 7,000 NPCs per well were added to each well of a non-coated 96-well plate. The following plates were tested: V-bottom plates (CELLSTAR 96-well V-shaped-bottom microplate, Greiner Bio-One, Catalog #GB651204), U-bottom plates (96-well clear round bottom ultra-low attachment microplate, ThermoFisher Scientific #50-211-3787), and Biofloat plates (BIOFLOAT™ 96-well plate #F202003, faCellitate). An additional plate (AggreWell 400 6-well plate, STEMCELL Technologies™, Catalog #34460) was included, as the microwell design could potentially facilitate a higher yield of MPS. AggreWell cultures were plated with a seeding density of 2.4×10^5^/well PMs and 5.6×10^5^/well NPCs per well. All co-cultures were gently pipetted up and down and maintained statically at 37 °C with 5% CO_2_ for up to 4 days in Neural Expansion Medium to form μbMPS.

To assess aggregation, 14 individual bMPS were randomly selected on day 4 and imaged with an EVOS XL Core Imaging System microscope (ThermoFisher Scientific). Size conformity was measured automatically using FIJI OrgM, and cellular processes radiating from the bMPS were manually counted by a blinded observer. The optimal plate was further assessed for inter-plate consistency by repeating the above experiment in triplicate.

For the remaining longitudinal and functional studies, U-bottom 96-well plates (ThermoFisher Scientific #50-211-3787) were selected, where PMs and NPCs were seeded in a 50:50 ratio with 1,000 PMs and 1,000 NPCs per well to form μbMPS^96-6^. NPCs-only controls containing 2,000 NPCs/well were used for comparison (bMPS^96-6^). After 3 days in 96-well plates with Neural Expansion Medium, all 96 μbMPS^96-6^ or bMPS^96-6^ cultures were transferred to a single well of a non-coated 6-well plate. The medium was then replaced with bMPS Differentiation Medium, and cultures were maintained under gyratory shaking (88 rpm, 19 mm orbit) for 9 weeks. These cultures were designated as *μ*bMPS^96-6^, where μ represents microglia inclusion, and 96-6 refers to their initial generation in 96-well plates followed by maintenance in 6-well plates. Unless otherwise noted in the figure caption, all experiments were conducted using a 50:50 ratio of NPCs and PMs.

### Terminology

In summary, we will refer to the following cultural conditions throughout the text:

**bMPS**^**6-6**^: Organoids were generated from 2×10^6^ NPCs per well of a 6-well plate (Romero et al., 2023) and contain no microglia.
**bMPS**^**96-6**^: Organoids were formed from 2,000 NPCs in a U-bottom 96-well plate first and then transferred to a 6-well plate and contain no microglia.
**μbMPS**^**96-6**^: Microglia containing assembloids were formed from 1,000 NPC and 1,000 PM in a U-bottom 96-well plate first and then transferred to a 6-well plate.
**bMPS**: A general term used when discussing brain microphysiologic systems broadly.

Organoids—These are used throughout the introduction and discussion sections to refer to published protocols that identify their model as organoids, in accordance with published nomenclature consensus for nervous system organoids (Pașca et al., 2022). bMPS is used in our study to include both neural organoids and microglia-containing assembloids.

### RNA extraction and qRT-PCR

bMPS were washed twice with ice-cold PBS, and all supernatant was removed before being snap-frozen in liquid nitrogen. Samples were stored at −80 °C before RNA extraction (Quick-RNA Miniprep Kit, Zymo Research, Catalog # R1054). RNA quantity and purity were determined using a NanoDrop 2000c (Thermo Fisher Scientific, Catalog # ND-2000C). 500 ng of RNA was reverse transcribed using the MLV Promega RT (Promega, Catalog #M1701) according to the manufacturer’s recommendations. Gene expression was evaluated using TaqMan^®^ Gene Expression Assays (Supplementary Table S1, Life Technologies) on a 7,500 Fast Real-Time system (Applied Biosystems). Fold changes were calculated with the 2^−ΔΔCt^ method (Schmittgen and Livak, 2001). *β-ACTB* and *18S* were used as housekeeping genes. Data are presented as mean ± SD, normalized to housekeeping genes and age-matched bMPS^6-6^.

### bMPS immunofluorescence

Samples were washed with ice-cold PBS and fixed with 4% paraformaldehyde in PBS. They were permeabilized with 0.05% Tween-20 and 0.05% Triton X-100 in PBS for 50 min on ice, followed by blocking with BlockAid for 1 h (Invitrogen, Catalog #B10710). Subsequently, samples were immunostained with primary antibodies (Supplementary Table S2) for 24–48 h at 4 °C with rotation. After three washes (1% Triton X-100, 0.5% BSA w/v in PBS), samples were incubated with secondary antibodies (bMPS were incubated overnight at 4 °C with rotation; monocultures were treated for 1 h at room temperature). Following three additional washes and staining with Hoechst33342, samples were transferred to glass microscope slides, mounted with ImmunMount (ThermoFisher Scientific, Catalog # 9990402), and imaged using LSM800 confocal microscope (63x Airyscan), LSM880 confocal microscope (63X and 20x), Olympus FVS3000R confocal microscope (100x and 20x), or Yokogawa C1Q dual-spinning disk High-content Imaging microscope (10X, 20X, and 63X), as indicated in the figure captions. Live-cell images were also obtained during medium changes throughout the culture period using an EVOS brightfield microscope. For 2D monoculture samples, cells were plated and fixed in 24-well glass-bottom plates with high-performance #1.5 coverslips (Cellvis, Catalog # P24-1.5H-N) and imaged using LSM800 (63X with Airyscan).

To perform the live-labeling of premature microglia, a 2.5 mg/mL stock solution of DiD’ oil (ThermoFisher Scientific, Catalog #D307) was prepared in 100% DMSO. At the time of labeling, 1 μL of DiD’ oil was mixed with 1 mL of cell suspension and incubated for 10 min at 37 °C. Following incubation, cells were pelleted at 302 g for 3.5 min, washed with Knockout DMEM (ThermoFisher Scientific, Catalog #10-829-018), and resuspended in Neural Expansion Medium. Labeled PMs were then combined with NPCs as described above. The labeled microglia were visualized live using far-red fluorescence imaging on a Yokogawa C1Q microscope maintained at 37 °C with 5% CO_2_.

### μbMPS^96-6^ dissociation

μbMPS with DiD’-oil-labeled microglia were fully dissociated as previously described (Romero et al., 2023; Morales Pantoja et al., 2024). Briefly, bMPS were washed twice with Hibernate-E (Thermofisher Scientific, Catalog #A1247601), then incubated one per well in a 24-well plate with Papain and DNase1 (Worthington Biochemical Corporation, CAS: 9035-91-1), prepared according to the manufacturer’s instructions, under gyratory shaking. The cells were gently pipetted every 30 min and returned to gyratory shaking until complete disassociation was achieved (approximately 4 h). The contents of each well were transferred to a 15-mL conical tube, centrifuged at 302 g for 4 min, and the supernatant removed. The pellet was resuspended in 1 mL Hibernate-E and incubated with 1 mL of ovomucoid-albumin (Worthington Biochemical, CAS: 9035-91-1), prepared according to the manufacturer’s instructions, to inactivate papain. Nuclei were labeled with Hoechst 33342 (ThermoFisher Scientific, Catalog #62249), and all cells were plated onto a single coverslip. Slides were imaged on a Yokogawa C1Q, and total cell number per organoid, as well as the percentage of DiD’-positive microglia, were quantified across three μbMPS.

### Image analysis

Synaptic density was evaluated per region of interest (ROI) (full image taken at 100x), with synapses identified by the colocalization of pre- and post-synaptic markers using the SynapseJ plugin in ImageJ (Moreno Manrique et al., 2021). Briefly, calibrated images were smoothed with a median blur, puncta were identified by thresholding and size exclusion, and ROIs without pre- and post-synaptic marker overlap were eliminated.

Microglia morphology was analyzed in ImageJ using the “Microglia Morphometry” plugin (Martinez et al., 2023). Briefly, calibrated images were smoothed and binarized using user-defined intensity thresholds. Individual microglia were then automatically segmented, and morphology parameters were quantified, including diameter and perimeter measurements, along with skeleton-based features such as average branch length, roundness, circularity versus elongation, and branch complexity.

### Functional assays

#### Lipopolysaccharide challenge and phagocytosis

LPS challenge and phagocytosis in microglia monolayer cultures: HPCs were plated on glass-bottom 24-well plates coated with PLO and laminin (Millipore Sigma, Catalog #A-0040C and #L2020), differentiated into PMs for 24 days, and then matured for an additional 8 days. pHrodo red *Escherichia coli* bioparticles (ThermoFisher Scientific, Catalog #P35361) were added to the plated cells according to manufacturer-recommended dilutions (1 mg/mL) for 3 h. Samples were fixed and immunostained as previously described. Images were captured with LSM800 at 63X magnification and analyzed using the microglia morphometry plugin in ImageJ (Martinez et al., 2023).

Lipopolysaccharide (LPS, ThermoFisher Scientific, Catalog #00-4976) stock (2.5 mg/mL) was sonicated for 1 min upon receipt, then aliquoted into single-use tubes, which were stored at −20 °C. For subsequent assays, aliquots were thawed, sonicated for 1 min, and then diluted in appropriate media (microglia maturation medium for monoculture assays and bMPS differentiation medium for bMPS assays). LPS was added directly to wells and incubated for 24 h at 37 °C under standard culture conditions.

Two phagocytosis assays were conducted in μbMPS^96-6^. In Experiment 1, μbMPS were divided into groups of 20 per well in brain organoid differentiation medium and exposed to LPS at 50, 200, and 500 ng/mL for 24 h. Phagocytosis particles were added at matching concentrations during the final 3 h of exposure (after 21 h). All dilutions were prepared in bMPS differentiation medium. Samples were harvested and processed for immunofluorescence immediately after fixation, as previously described. In Experiment 2, pHrodo Red *E. coli* bioparticles were added to μbMPS^96-6^ at 1 mg/mL (per the manufacturer’s recommendations) and incubated in a stationary position at 37 °C for 3 h, followed by harvesting and immunofluorescence processing immediately, as previously described.

### Calcium imaging

Calcium imaging was performed as previously described (Alam El Din et al., 2025). Briefly, samples were placed at 37 °C and 5% CO_2_ without shaking and incubated with 10 μM Fluo-4 AM (Tocris, Catalog # 6255) and 0.5% Pluronic Acid (Invitrogen, Catalog # P3000MP) for 2 h. Samples were then washed with differentiation medium three times and placed in a glass-bottom dish for live imaging. The samples were imaged using an Olympus FV3000-RS at a speed of 12.5 frames per second for 6 min while maintaining a temperature of 37 °C and 5% CO_2_ throughout. The data were processed and quantified as previously described (Alam El Din et al., 2025).

### High-Density Micro-Electrode Array

Samples were plated and recorded as previously described (Alam El Din et al., 2025). Briefly, both MaxWell Biosystems 6- and/or 24-well HD-MEA chips were coated for 1 h at 37 °C with 5% CO_2_ using a 0.07% Poly(ethyleneimine) solution (Millipore Sigma catalog # 03880) diluted in 1x borate buffer (ThermoFisher Scientific, Catalog # 28341). The HD-MEA chips were then washed three times with water and dried in a sterile hood for 1 h. A solution of 0.04 mg/mL mouse laminin was diluted in differentiation medium and applied to the chips for overnight incubation at 37 °C with 5% CO_2_. Afterwards, laminin was removed, and samples were placed on the MEA in differentiation medium, with one bMPS per well. Breathe-EASY membranes (Millipore Sigma, Catalog #Z380059) were used to seal the HD-MEA plates, prevent evaporation, and maintain humidity in the wells. Day on MEA 0 (DOM 0) is defined as the day samples were placed on the chip. The MaxWell Biosystems MaxLab Live Software (version 22.2.6, Switzerland) was used to calculate percent active area, interspike interval within bursts, burst interspike interval, interburst interval coefficient of variation, burst peak firing rate, spikes per burst per electrode, spikes per burst, spikes within bursts, burst frequency, burst duration, and interspike interval outside bursts. Finally, a gain of 512x, a high-pass filter of 300 Hz, and a spike threshold of 5.5 RMS mV were used for all recordings.

### Cytokine measurement

To evaluate secreted cytokines, we collected 50 μL of supernatant twice per well at the end of exposures, yielding two technical replicates per biologic replicate, in accordance with the manufacturer’s recommendations. Supernatants were frozen at −80 °C in 96-well plates (Falcon, Catalog #353072) sealed with adhesive aluminum foil (ThermoFisher Scientific, Catalog #AB0626) until measurement. Although the Curiox DropArray miniaturization step requires only 10 μL of supernatant, 50 μL of supernatant was collected and stored at −80 °C to facilitate pipetting and minimize the risk of sample evaporation. On the day of the assay, samples were thawed and mixed by pipetting up and down 8–10 times before the assay. Cytokines were evaluated using the Luminex 25-plex human cytokine panel (ThermoFisher Scientific, LHC0009M) and the Luminex 30-plex human cytokine panel (LHC6003M, Supplementary Table S3), miniaturized with Curiox DropArray technology. Briefly, Curiox DropArray plates were blocked with 1% BSA, followed by three Curiox washes. All washes were performed with 0.5% BSA and 0.05% Tween-20 in PBS. Subsequently, 10 μL of Luminex beads were added to each well, and the plates were kept on magnet arrays throughout all subsequent steps. Standards were prepared using 1:4 serial dilutions of the provided standard solutions. Then, 10 μL of sample supernatant was added to each well, with biological triplicates and technical duplicates, according to the manufacturer’s recommendations. Samples were incubated on a shaker at 4 °C overnight, followed by three Curiox washes. Finally, samples were incubated for 1 h with biotinylated antibody and for 1 h with streptavidin detection antibody, with three washes between each step. Samples were removed from the magnet array, vigorously pipetted with Magpix drive fluid to collect Luminex beads, and transferred to a hard-walled PCR plate (ThermoFisher Scientific, Catalog#AB-0800) for analysis on a Luminex Magpix machine.

Then, 5 pL regression curves were generated from standard curves independently for each cytokine, with standard curves determined per plate to account for inter-plate variability. Extrapolated values beyond the standard ULOQ and LLOQ were accepted. Values below the range were adjusted to be LLOQ or the minimum extrapolated value/SQRT (Matejuk and Ransohoff, 2020). Values above the range were adjusted to the ULOQ or to the maximum extrapolated value*1.5 (Ortega-Villa et al., 2021). Technical replicates were averaged, and cytokine concentrations were normalized to unexposed controls within each group (bMPS^96-6^ or μbMPS^96-6^).

### Traumatic brain injury model

The traumatic brain injury (TBI) model was adapted from a previously published protocol (González-Cruz et al., 2024). Eight-week μbMPS^96-6^ were randomly split into three groups: (1) TBI-challenged (*n* = 16), (2) no-TBI controls (*n* = 16), and (3) time 0 controls (*n* = 4). For TBI induction, μbMPS^96-6^ were transferred to microcentrifuge tubes containing 1 mL of medium, centrifuged at 2,862 g-force for 4 min at room temperature, then returned to a 6-well plate on a gyratory shaker and incubated for 4 and 24 h. Batch-matched controls were transferred to a microcentrifuge tube, left static for 4 min at room temperature, and then returned to a 6-well plate on a gyratory shaker and incubated for 4 and 24 h. Time 0 controls were fixed before any manipulations. All cultures were fixed, stained as described above, and imaged using an Olympus FVS3000R microscope.

### RNA sequencing

Two-week-old μbMPS were washed with ice-cold PBS and then snap-frozen in liquid nitrogen. RNA extraction was performed as described above (for qPCR) in triplicate. Samples were transferred to the Johns Hopkins Genomics Core for library preparation and Illumina sequencing. mRNA libraries were constructed by the core facility following the manufacturer’s recommended protocol with the NEBNext^®^ RNA Library Prep Kit for Illumina sequencing. Samples were then multiplexed and run on a NovaSeq 6,000 instrument at the Johns Hopkins Genomics Core Facility to generate 50 million 150-base pair paired-end reads per sample.

Raw FASTQ data were aligned using the Nextflow nf-core rnaseq pipeline (version 23.10.1) to the GRCh38 genome with ENSEMBL gene annotations (release 111) (Ewels et al., 2020; Martin et al., 2023). Poor-quality sequences and adapters were removed using “Trim Galore!” (Krueger et al., 2023). Transcripts were aligned with STAR and quantified using Salmon (Dobin et al., 2013; Patro et al., 2017). Sorting and indexing of reads were performed with SAMtools, duplicates were identified with Picard, and genome coverage was assessed with BEDtools (Quinlan and Hall, 2010; Li et al., 2009; Picard Toolkit, 2019). RSeQC, Qualimap, dupRadar, and Preseq were used to determine read and mapping quality (Wang et al., 2012; García-Alcalde et al., 2012; Sayols et al., 2016; Computational Genomics Research, 2019). Expression data were normalized using TMM (weighted trimmed mean of M-values) with the tidybulk package and subsequently log2-transformed (Mangiola et al., 2021). Known microglia marker genes were selected from published databases (Miller-Rhodes, 2022), and log2 fold changes were plotted for individual replicates.

### Statistical analysis

Statistical analysis was conducted using GraphPad Prism (Version 10). All data were evaluated for normality, outliers, and homoscedasticity, and non-parametric analyses were utilized when appropriate. Optimizations to select plates included area conformity, the number of processes of μbMPS, and inter-plate consistency, which were assessed by Kruskal-Wallis with post-hoc Dunn’s test. In the longitudinal assessment, qRT-PCR results were analyzed by the Kruskal-Wallis test, and bMPS size over time was evaluated with a two-way ANOVA and repeated Tukey’s multiple comparisons test. Image analysis, when quantified, and cytokine measurements were assessed with ordinary one-way ANOVA with subsequent Dunnett’s multiple comparisons to the control group. A statistically significant difference in synaptic density was evaluated by the Kolmogorov–Smirnov test. An adjusted *p*-value of <0.05 was considered statistically significant.

### Literature review

A non-scoping literature review was performed using a Google Scholar search with the terms “microglia” AND “organoid” OR “assembloid” OR “microphysiologic system.” Titles were reviewed, and all articles discussing brain or neural organoid systems were selected for further consideration, with a subsequent review of all articles’ abstracts to identify those characterizing a 3D, microglia-inclusive cell culture model (excluding retinal organoids).

Publications were briefly reviewed to exclude documents that were subsequent publications based on already published and described microglia-containing models. Models derived from brain tumor resection or human brain slices were also excluded. Xenotransplantation models were only included if they had an entirely *in vitro* characterization. Only peer-reviewed publications were included.

Articles were then reviewed in full, with particular attention to the source of microglia (iPSC or other source), whether the organoid media was altered in any way from the standard organoid media used by the authors’ non-microglia model, whether the microglia were present from the formation of organoids, and whether long-term survival was demonstrated in the main figures (long-term considered to be ≥75% of the culture period). Venn diagrams were generated with the publicly available tool jvenn (Bardou et al., 2014).

## Results

### hiPSC-derived microglia culture exhibits expression of mature microglia markers and activation potential

**Figure 1:**
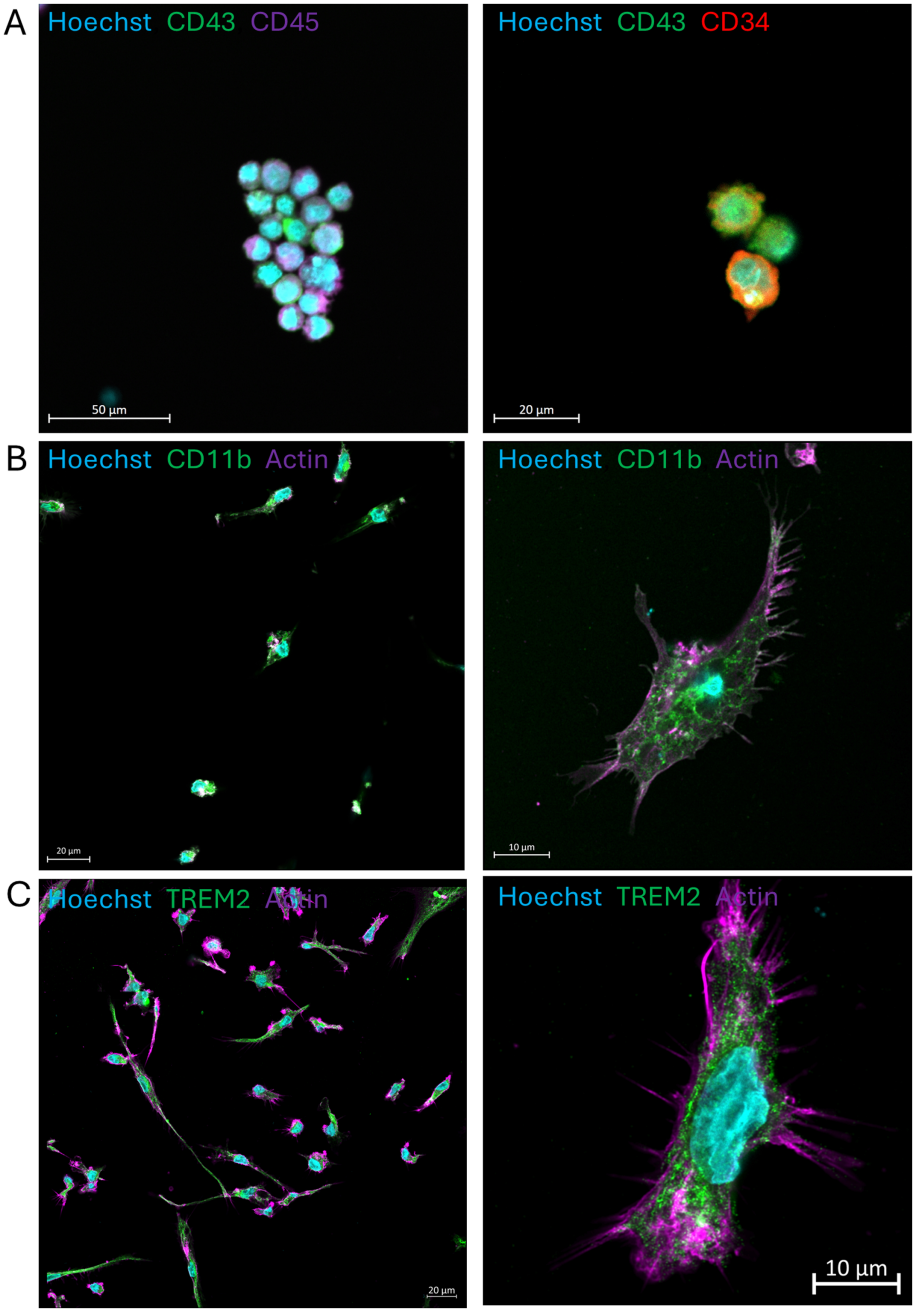
iPSC-derived microglia differentiated and matured in a monolayer. **(A)** hiPSC-derived hematopoietic precursors collected after 12 days of differentiation and stained with antibodies against CD43, CD45, and CD34. **(B,C)** Mature microglia show positive staining for CD11b and TREM2. Nuclei are stained with Hoechst 33342. Scale bars are 50, 20, and 10 μm. Images were taken on the LSM800 with Airyscan.

First, we confirmed the proper differentiation of NIBSC8 iPSCs into mature microglia in monolayer cultures following the STEMCELL Technologies protocols for hematopoietic differentiation, microglia differentiation, and microglia maturation. NIBSC8 hiPSCs differentiated robustly into CD34/43/45-positive hematopoietic precursor cells (Figure 1A) and a highly pure population of microglia cells that expressed mature and specific microglia markers CD11b and Trem2 (Figures 1B,C).

**Figure 2:**
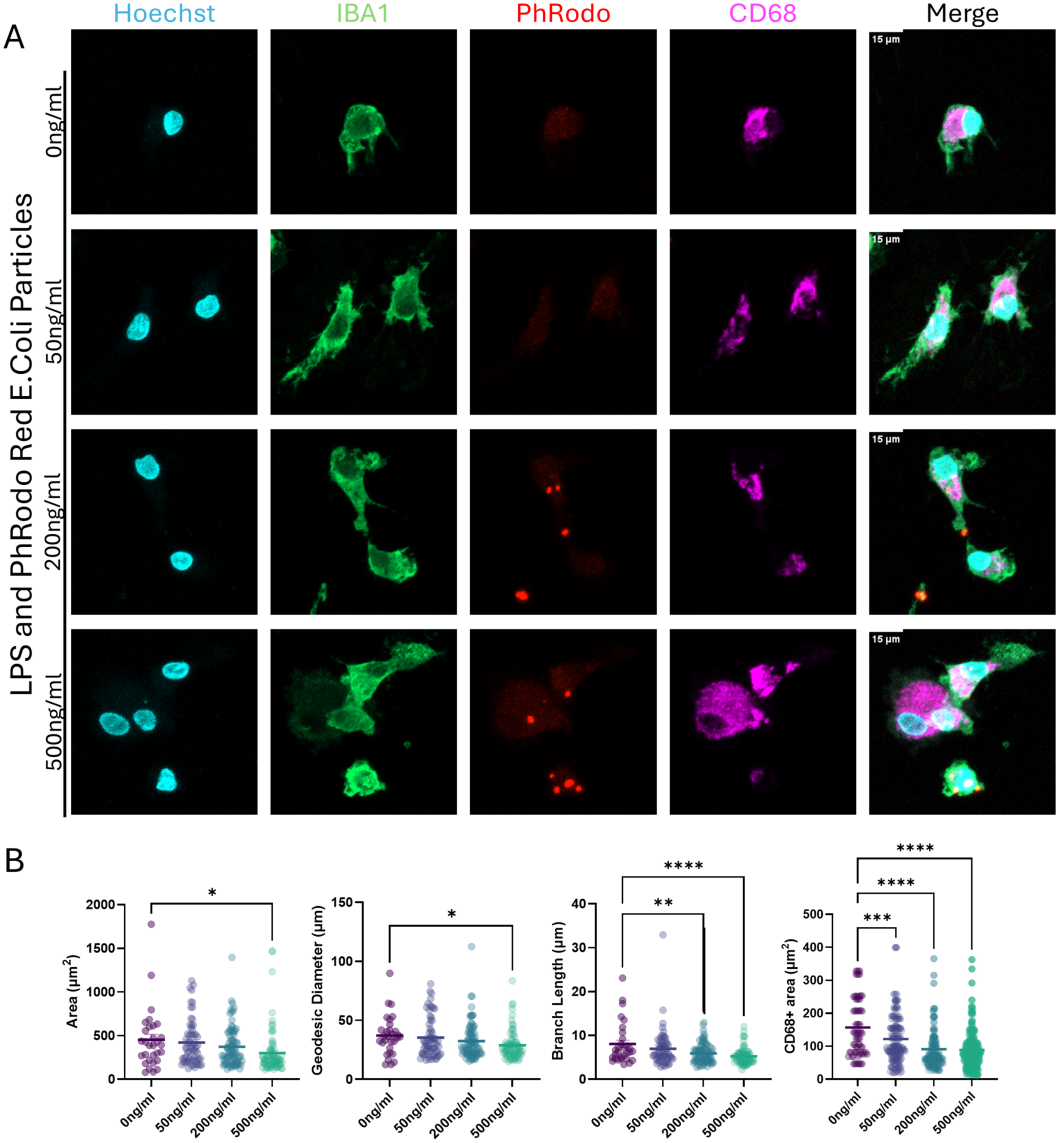
Activation of microglia differentiated and matured in a monolayer with lipopolysaccharide (LPS); microglia phagocytosis capabilities. **(A)** Mature microglia exposed to LPS at increasing concentrations (0, 50, 200, and 500 ng/mL) for 24 h, with pHrodo red *E. coli* bioparticles added during the final 3 h of incubation. Cells were labeled with Hoechst 33342 (blue, nuclei), IBA1 (green, cytoplasm), pHrodo red *E. coli* bioparticles, and CD68 (magenta, lysosomal marker). The scale bar is 15 μm. **(B)** Microglia morphology quantifications are based on cell area, diameter (geodesic), skeleton branching length, and area of CD68^+^ signal. Each dot represents an individual cell analyzed. The bar represents the mean from all cells quantified. Statistical significance was analyzed by one-way ANOVA with subsequent Dunnett’s multiple comparisons, corrected for * *p* < 0.05, ** *p* < 0.01, *** *p* < 0.001, **** *p* < 0.0001. Microglia were imaged on LSM800 with Airyscan.

To confirm that the microglia cells were capable of phagocytosis and could be activated, we challenged them with lipopolysaccharide (LPS) and fluorescently labeled *E. coli* bioparticles at 0, 50, 200, and 500 ng/mL. *In vitro* exposures to LPS are not an appropriate model for clinical sepsis; however, these doses are within the range used to induce inflammation in other models and are non-cytotoxic (Abreu et al., 2018; Vasilescu et al., 2009; Yücel et al., 2017; Honda and Inagawa, 2023). The pHrodo red *E. coli* bioparticles contain pH-sensitive fluorescence, thus increasing their fluorescence when internalized by cells into acidic compartments such as the phagolysosome. We demonstrated that microglia cells are capable of phagocytosing bioparticles (Figure 2A). The cells were responsive to LPS in a concentration-dependent manner, showing trends toward ameboid morphology with decreased cell mask area, decreased skeletal branch length, and decreased CD68^+^ (microglial lysosomal or endosomal marker) area, consistent with a trend toward ameboid morphologies (Figure 2B).

### Optimization of microglia integration into the bMPS

To achieve our goal of developing a model that allows for a controllable ratio of microglia within the organoids, which is both consistent and reproducible, we assessed three separate integration methods to identify the most robust technique. Briefly, the three methods are as follows: (1) adding premature microglia to 3-day post-generation bMPS; (2) combining neuronal progenitors and premature microglia directly in a 6-well plate; and (3) adding neuronal progenitors and premature microglia to individual wells of a 96-well plate, allowing aggregation, and then transferring the resulting μbMPS to a 6-well plate (schematized in Supplementary Figure S2A).

To assess integration efficiency, all bMPS from a well were immunostained with the microglia marker IBA1 and the neuronal marker NF200, and the percentage of aggregates containing IBA1^+^ cells was manually counted by a blinded observer. Integration technique 3, which combines PM and NPCs in a 96-well plate before transferring to a 6-well plate on a shaker, was found to be the most robust technique, with 85.7% of bMPS containing microglia. Integration techniques 1 and 2 both resulted in PMs present as single cells in suspension or having formed PM-only spheres, and microglia were visible in only 5.6% or 0% of bMPS analyzed, respectively. Since integration technique 3 allowed precise control of the cell number of integrated microglia with a high level of successful integration, we followed this technique for the subsequent experiments.

### U-bottom 96-well plates are optimal for the generation of standardized microglia containing bMPS

**Figure 3:**
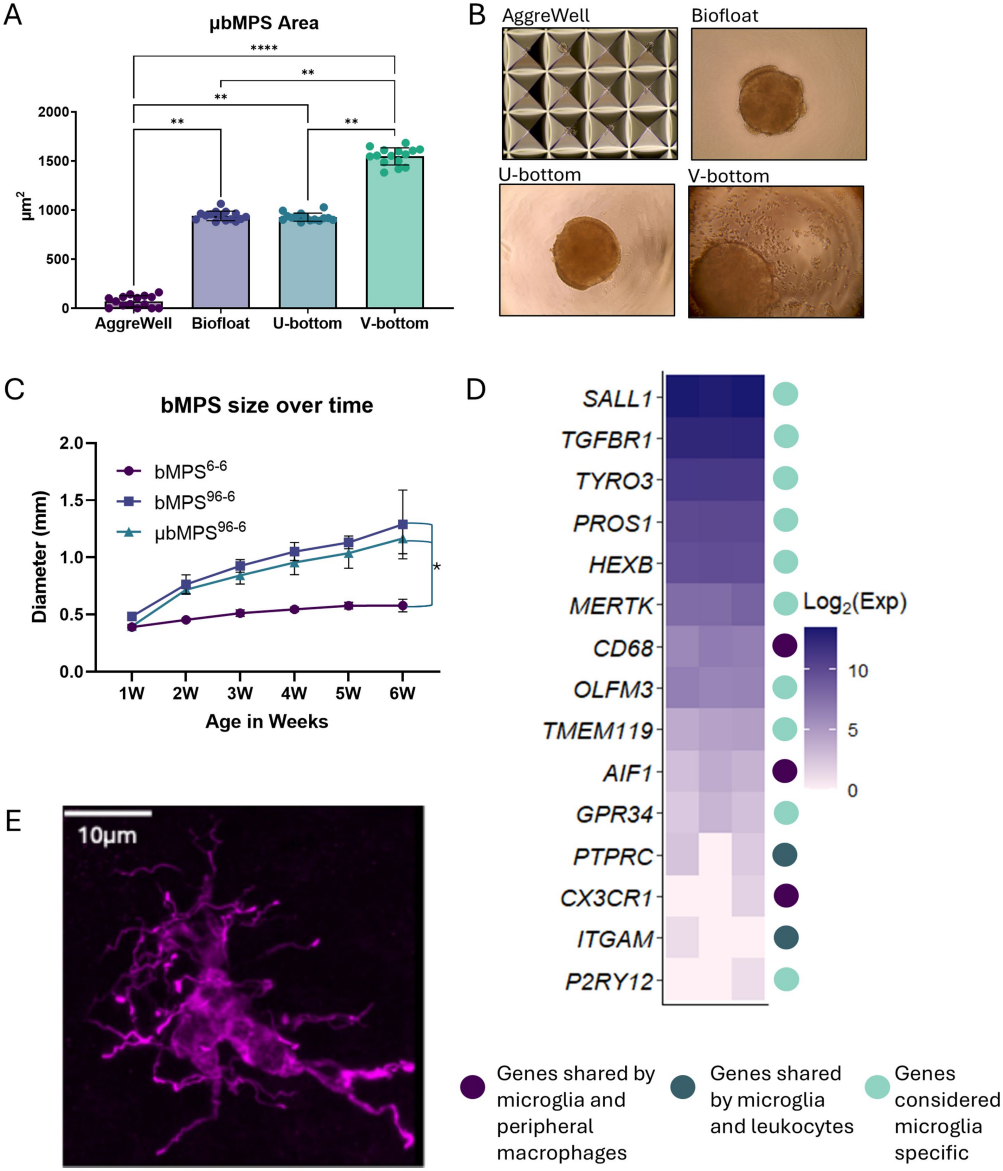
Optimization of the initial aggregation of NPCs and PMs in different plate formats. **(A)** Areas of individual bMPS generated on four different plates. Data were evaluated using Kruskal-Wallis and post-hoc Dunn’s test * *p* < 0.05, ** *p* < 0.01, *** *p* < 0.001, **** *p* < 0.0001. **(B)** Representative images of aggregation in each plate type. **(C)** bMPS^6-6^, bMPS^96-6^, and μbMPS^96-6^ size over 6 weeks in culture. bMPSs were established from the same batch of NPCs and cultured in parallel; 10–15 bMPSs per condition were assessed at each time point. Data were analyzed with two-way ANOVA and Tukey’s pairwise comparisons at each time point, showing a significant difference between bMPS^6-6^ and bMPS^96-6^ starting at 2 weeks post-integration, with no significant differences due to the presence of microglia, * *p* < 0.05. **(D)** Log_2_(expression) changes in microglia-related genes from bulk RNA sequencing of 2-week-old μbMPS^96-6^, with color coding indicating gene specificity. **(E)** Example of integrated IBA-1 positive ramified microglia in the 8-week-old organoid Scale bar - 10 μm.

To assess the impact of plate type on culture size and conformity, different 96-well plate formats were tested using increased cell densities to facilitate rapid evaluation. 3,000 PMs and 7,000 NPCs were seeded into V-bottom, U-bottom, and Biofloat plates, while a microwell-based AggreWell plate was also evaluated for its potential to enhance bMPS yield and throughput (Figure 3; Supplementary Figure S2B). Across the different plates assessed, μbMPS area and conformity varied significantly (Figure 3A). AggreWell plates failed to enable μbMPS formation of a noticeable size. V-bottom plates generated the largest and most variably sized μbMPS. Only U-bottom plates and Biofloat plates produced μbMPS with reproducible area (*p* > 0.999); however, there was higher inter-μbMPS consistency with U-bottom plates (Figure 3A). Additionally, some μbMPS were observed to have attached to the biofloat plates or have cellular protrusions (Figure 3B and Supplementary Figure S2B).

Assessment of inter-plate consistency across three independent experiments using U-bottom plates showed no significant differences in μbMPS size between plates (*p* = 0.83; Supplementary Figure S2C). However, using 10,000 cells per well produced exceptionally large organoids (more than 1 mm in diameter at 2 weeks). To address this, the total seeding number of NPCs and PMs was reduced to 2,000 cells per well, which decreased organoid size (approximately 0.7 mm diameter at 2 weeks) and improved μbMPS yield per batch of premature microglia. The protocol was also refined by reducing the aggregation time in a 96-well plate from 4 to 3 days. This adjustment eliminated the need for a medium change after 48 h and limited organoid size, as NPCs proliferate rapidly in Neural Expansion Medium.

Finally, a 7:3 ratio of NPCs to PMs was initially selected based on Xu et al. (2021). However, due to the rapid proliferation of NPCs, we found that a final ratio of 50:50 was more appropriate for an optimal microglia-to-neuron ratio in the mature μbMPS. With these refinements of the protocol, 100% of μbMPS^96-6^ had IBA1^+^ microglia visible when analyzed at 2 weeks post-integration.

Overall, due to the improved uniformity and consistency of μbMPS^96-6^ in U-bottom plates—along with minimal attachment or process formation—this protocol was used for all subsequent experiments, with 2,000 cells per well at a 50:50 PM:NPC ratio, 3 days of aggregation in 96-well plates and subsequent culture in a 6-well plate for up to 9 weeks.

We generated bMPS with and without PMs in U-bottom 96-well plates (μbMPS^96-6^ and bMPS^96-6^, respectively), alongside the standard protocol (bMPS^6-6^), to evaluate whether the inclusion of premature microglia impacted bMPS growth and size and to compare it with our standard protocol over time. We quantified the size of these three cultures over 6 weeks of differentiation. bMPS^96-6^ were significantly larger than bMPS^6-6^ as early as 2 weeks after integration and continued to grow in size throughout the culture period, with no significant differences due to the presence of microglia (Figure 3C).

To confirm that the differentiation produces cells resembling a microglia lineage, we performed bulk RNA sequencing at 2 weeks post-integration. We identified a panel of microglia-specific or microglia-related genes from the literature (Miller-Rhodes, 2022) and evaluated their expression in these samples. Although the relative expression of some markers was low, we observed an enrichment of many microglia-specific genes, such as *SAL1* or *HEXB* (Figure 3D). Although single-cell sequencing would more effectively confirm that we have a homogenous population of microglia, these data are suggestive of microglia, rather than macrophage, fate.

Finally, a representative image of a microglia in an 8-week-old μbMPS demonstrates the highly ramified morphologies typically observed, consistent with a homeostatic state (Figure 3E).

### Microglia integrate into bMPS and survive for at least 9 weeks after integration

**Figure 4:**
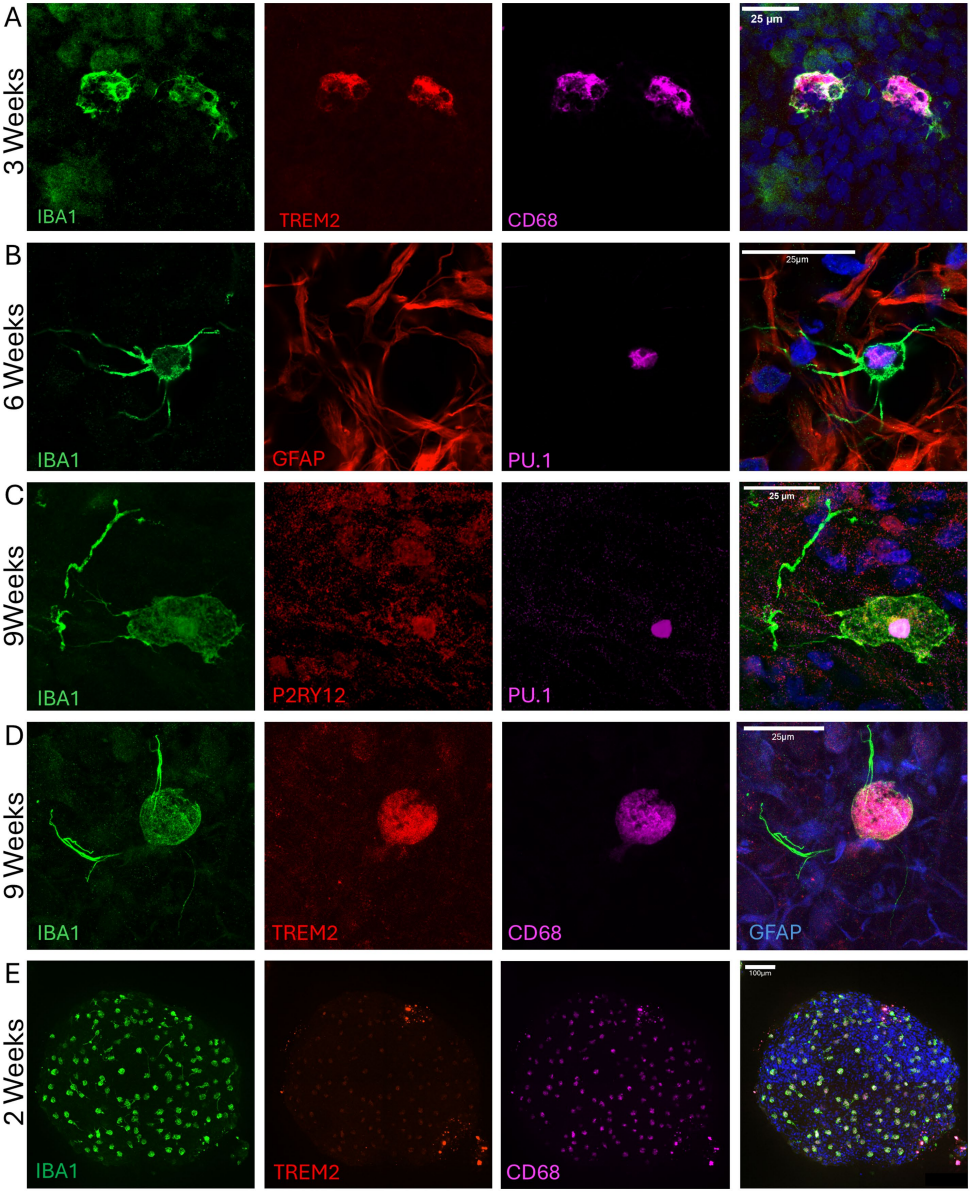
Microglia incorporation and long-term survival in organoids for up to 9 weeks. **(A)** Ramified microglia at 3 weeks post-integration expressing IBA1 (green), TREM2 (red), and CD68 (magenta). Nuclei are stained with Hoechst 33342 (blue). **(B)** Ramified microglia positive for IBA1 (green) and PU.1 (magenta) at 6 weeks post-integration. Astrocytes are stained with GFAP (red), and nuclei with Hoechst 33342 (blue). Presence of microglia with ameboid **(C)** and ramified **(D)** morphology at 9 weeks post-integration, expressing IBA1 (green), P2RY12 (red), TREM2 (red), PU.1 (magenta), and CD68 (magenta). In C, nuclei are stained with Hoechst 33342, while in D, astrocytes are co-stained with GFAP (blue). Nuclear P2RY12 staining in an ameboid cell indicates an inflammatory state. Images were taken with Airyscan on the LSM800 microscope and represent four independent experiments. **(E)** A representative low-magnification image of a 2-week-old whole μbMPS^96-6^ showing the relative abundance and distribution of microglia across the entire μbMPS; images taken on Yokogawa C1Q 20X tiled.

To validate integration, maturation, and survival during bMPS differentiation, we harvested μbMPS^96-6^ at 3, 6, and 9 weeks post-integration and immunostained with microglia markers (CD68, IBA1, PU.1, TREM2, and P2RY12) (Figures 3E, 4). We demonstrated that microglia are evenly distributed in the μbMPS^96-6^ at 2 and 6 weeks (Figure 4E, Supplementary Figure S3B and Supplementary video 1); we observed persistent survival of microglia in the μbMPS^96-6^ for at least 9 weeks, and microglia can adopt ramified morphologies consistent with a non-diseased state and healthy surveillance of the environment (Figures 3E, 4A,B,D). Microglia have also been identified with ameboid (Figure 4C) morphologies, suggestive of an inflammatory response.

### bMPS grown following the protocol with the 96-well plate integration step are overall larger than those generated in 6-well plates, but it does not affect neural cellular composition

**Figure 5:**
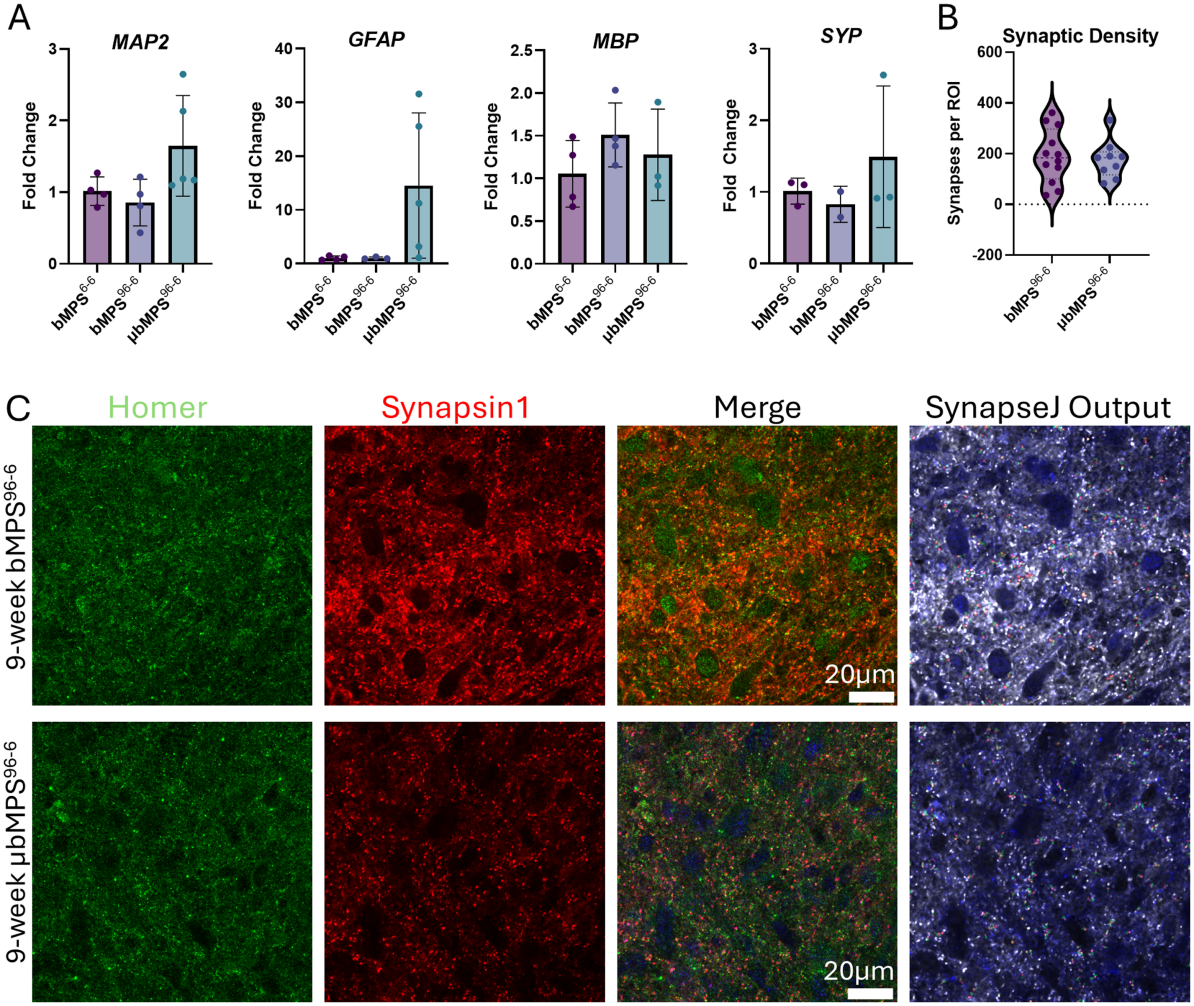
Gene expression of neural markers in μbMPS^96-6^ vs. bMPS^96-6^ and bMPS^6-6^, 8 weeks after integration. **(A)** *MAP2* (microtubule-associated protein, a marker of mature neurons and dendrites), *GFAP* (glial fibrillary acidic protein, a marker for astrocytes), *MBP* (myelin basic protein, a marker for oligodendrocytes), and *SYP* (synaptophysin, a pre-synapse marker). For μbMPS^96-6^ 7:3, the NPCs/PMs ratio was used for this experiment. Data represent mean±SD from two independent experiments, with four biological replicates in total, normalized to gene expression in bMPS^6-6^ samples. Statistical significance was evaluated using one-way ANOVA with Tukey’s multiple comparison tests. **(B)** Synaptic density of bMPS^96-6^ and μbMPS^96-6^. Synapses were identified through co-staining and the overlap of pre-(synapsin1) and post-(homer1) synaptic density markers imaged at 100x (Olympus FVS3000R microscope with resonant scanning) and analyzed with SynapseJ. Data represent synapse counts from three regions of interest (ROI) per organoid, with four organoids per condition. Significance was analyzed with Welch’s *t*-test. **(C)** Representative images used for **(B)**. The scale bar is 20 μm.

We then assessed the neural cellular composition in μbMPS^96-6^, bMPS^96-6^, and bMPS^6-6^ to evaluate whether the culture technique and/or the presence of microglia influence the differentiation efficiency of the main neural lineages. During 8 weeks of differentiation, there were no statistically significant differences in the expression of neuronal (*MAP2*) and oligodendrocyte (*MBP*) markers across the conditions, as measured by RT-qPCR. Notably, we observed a higher expression of *GFAP* in μbMPS^96-6^, although it did not reach statistical significance (Figure 5A).

Additionally, we quantified the expression of the synaptic marker (synaptophysin, *SYP*) in 8-week μbMPS^96-6^, bMPS^96-6^ vs. bMPS^6-6^, and detected no difference between the conditions (Figure 5A). We then analyzed synaptic density in 9-week-old μbMPS^96-6^ and bMPS^96-6^ by co-staining and colocalization of pre- and post-synaptic markers (Synapsin1 and Homer1). Consistent with qRT-PCR, we identified no statistically significant differences in synaptic density between bMPS^96-6^ and μbMPS^96-6^ (Figures 5B,C).

**Figure 6:**
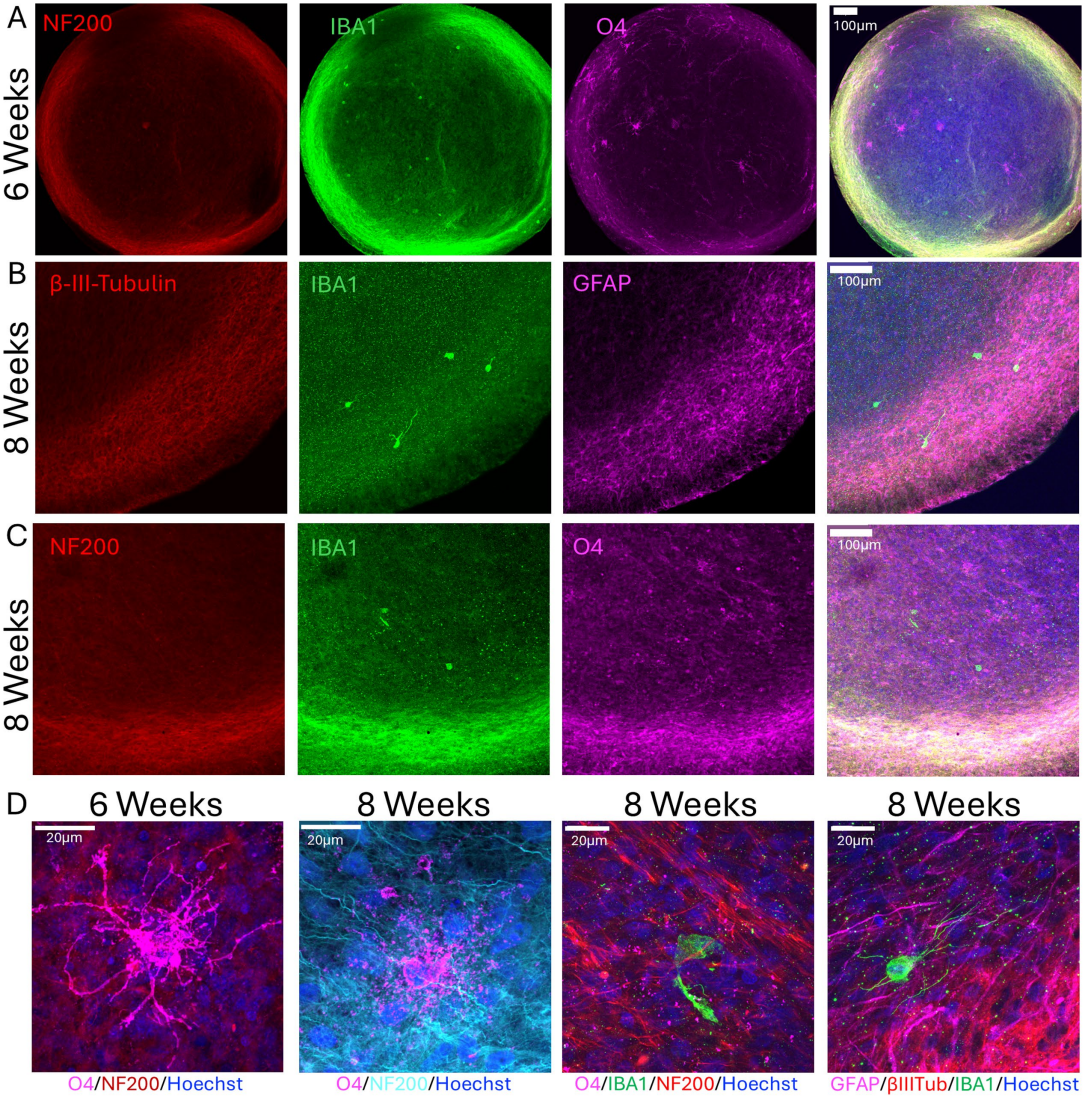
Presence of all main neural cell types in 6- and 8-week-old μbMPS^96-6^. **(A)** Six-week μbMPS^96-6^ stained with antibodies against NF200 (red), IBA1 (green), and O4 (magenta). **(B)** Eight-week μbMPS^96-6^ stained with antibodies against neurons (*β*-III-Tubulin, red), astrocytes (GFAP, purple), and microglia (IBA1, green). Nuclei are stained with Hoechst 33342. **(C)** The same staining as in A, but in 8-week-old organoids. **(D)** Higher-magnification images of 6- and 8-week-old μbMPS^96-6^ stained with antibodies against NF200, O4, IBA1, GFAP, and β-III-Tubulin. Images were taken with an Olympus FVS30004 microscope.

In addition, we stained μbMPS^96-6^ with neuronal (NF200, *β*-III-Tubulin), astrocyte (GFAP), and oligodendrocyte (O4) markers at 6 and 8 weeks of differentiation to demonstrate the presence of microglia and all main neural cell types in the mature μbMPS^96-6^. The presence of the expected morphologies of astrocytes and oligodendrocytes appeared as early as 6 weeks (Figures 6A–D).

To quantify the proportion of microglia in the μbMPS^96-6^, we labeled the microglia with a lipophilic dye before integrating them into the organoids. We dissociated 6-week-old μbMPS^96-6^ and counted the total number of nuclei, which averaged 1.9×10^5^ cells per organoid. The total number of microglia per organoid was on average 2.5% (1.34–3.5%) (Supplementary Figure S3A). In a previously published analysis of our bMPS^6-6^ model, we quantified the proportions of other cell types (Romero et al., 2023). Therefore, we expect the relative cell proportions to be approximately 35% astrocytes, 20% oligodendrocytes, 50% neurons, and 2.5% microglia at a 6-week timepoint, but single cell RNA sequencing is needed to estimate the percentage of different lineages with higher precision.

### Presence of microglia boosted spontaneous electrical activity in μbMPS^96-6^

**Figure 7:**
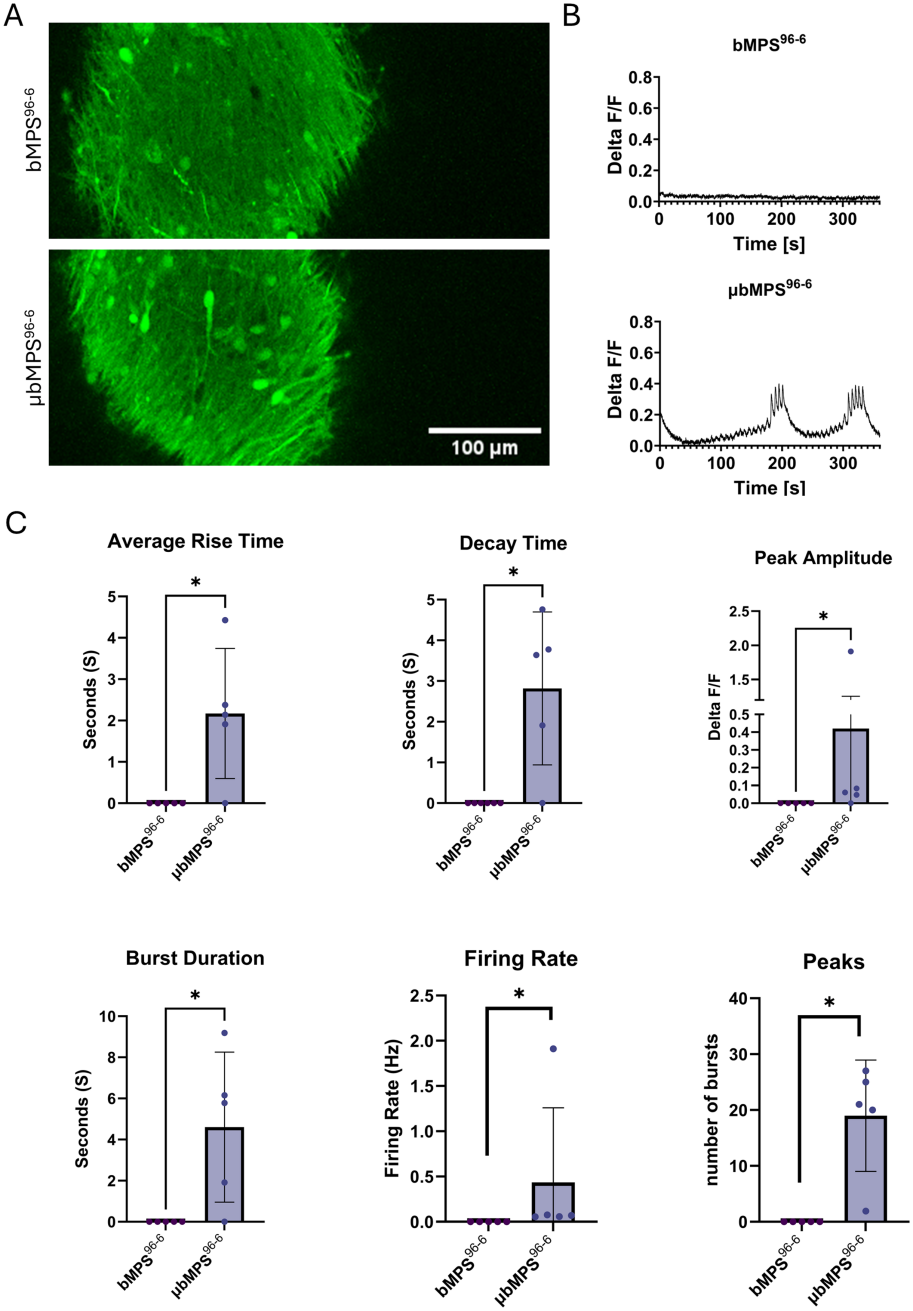
Ca^2+^-imaging in 3-week-old bMPS^96-6^ with and without microglia, measuring Ca^2+^ flux. Representative images **(A)** and Ca^2+^ traces **(B)**. **(C)** Quantification of Ca^2+^ traces: average rise time, decay time, and peak amplitude. Four bMPS were imaged per condition. Data were evaluated with the Mann–Whitney test, * *p* < 0.05.

Ultimately, recognizing the importance of microglia in neuronal network maturation and electrical activity, we characterized the spontaneous electrical activity of microglia-containing bMPS vs. those without microglia. Historically, bMPS^6-6^ exhibit no or very low spontaneous network activity detectable with calcium imaging at 3 weeks of maturation (Alam El Din et al., 2025). bMPS^96-6^ similarly does not exhibit spontaneous calcium potentials at week 3; however, μbMPS^96-6^ has clear network bursting patterns (Figure 7) with representative frames (Figure 7A) and traces (Figure 7B) shown. The bursts were characterized by average rise times, decay times, peak amplitudes, burst durations, firing rates, and the number of peaks observed in the recording period (Figure 7C). This suggests that microglia might contribute to the maturation of neural networks *in vitro*, as they do *in vivo* (Arnoux and Audinat, 2015).

**Figure 8:**
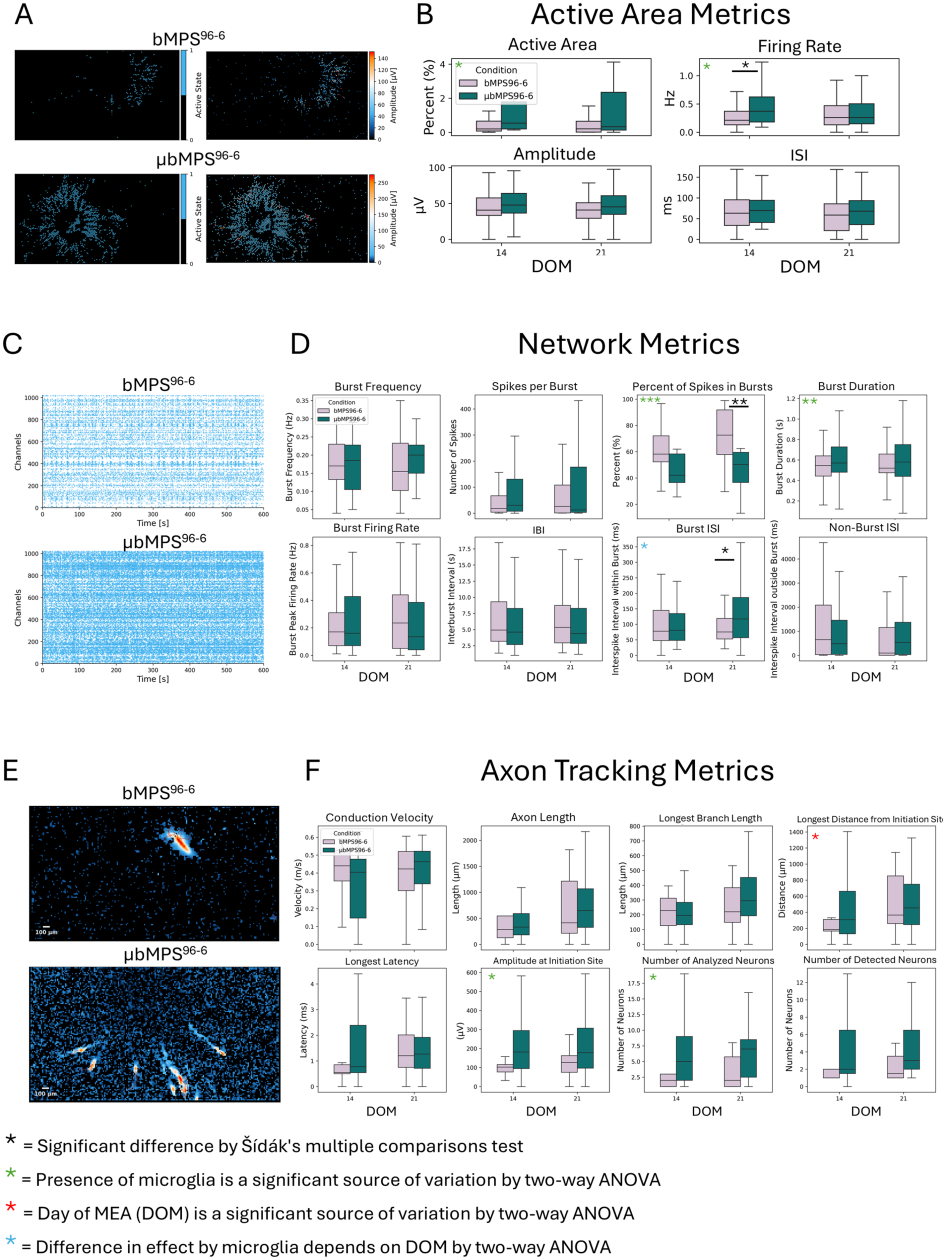
Spontaneous electrical activity in μbMPS^96-6^ vs. bMPS^96-6^ measured on HD-MEA at 14 and 21 days after plating. **(A)** Representative active area maps of μbMPS^96-6^ and bMPS^96-6^. **(B)** Quantified active area, firing rate, amplitude, and interspike interval at two time points: 14 and 21 days on MEA. **(C)** Representative raster plots of both cultures. **(D)** Quantifications of network metrics between μbMPS^96-6^ and bMPS^96-6^. **(E)** Representative plots of axon tracking analysis. **(F)** The quantified axon tracking metrics. Data were collected from three independent experiments with a total of 22 individual organoids per condition. Statistical significance was evaluated with a two-way ANOVA and Sidak’s multiple comparison test, *p < 0.05.

Then, we placed the bMPS^96-6^ and μbMPS^96-6^ on a High-Density Micro-Electrode Array (HD-MEA). We assessed active area metrics (including the percentage of active area, firing rate, amplitude, and inter-spike intervals), network metrics (characterizing spiking and bursting activity), and axon tracking in both conditions. Consistent with the calcium flux data, μbMPS^96-6^ showed an overall trend toward increased spontaneous electrical activity relative to bMPS^96-6^ (Figure 8). Active area metrics were significantly higher in μbMPS^96-6^ (Figures 8A,B), as were the firing rate (Figure 8B), the percentage of spikes in bursts, burst duration, and burst interspike interval (Figures 8C,D). These changes are also evident in graphical formats, raster plots, and quantifications.

Additionally, at the earlier time point of differentiation, the active axons were longer, as represented by the length from the initiation site, which was significantly increased (Figure 8F). Overall, this data demonstrated that bMPS containing microglia were more active (based on both active area and burst metrics), which is consistent with the calcium flux data. Future studies will help to elucidate the mechanisms through which microglia contribute to earlier network activity and maturity.

### Microglia within the bMPS were responsive to stress challenge

**Figure 9:**
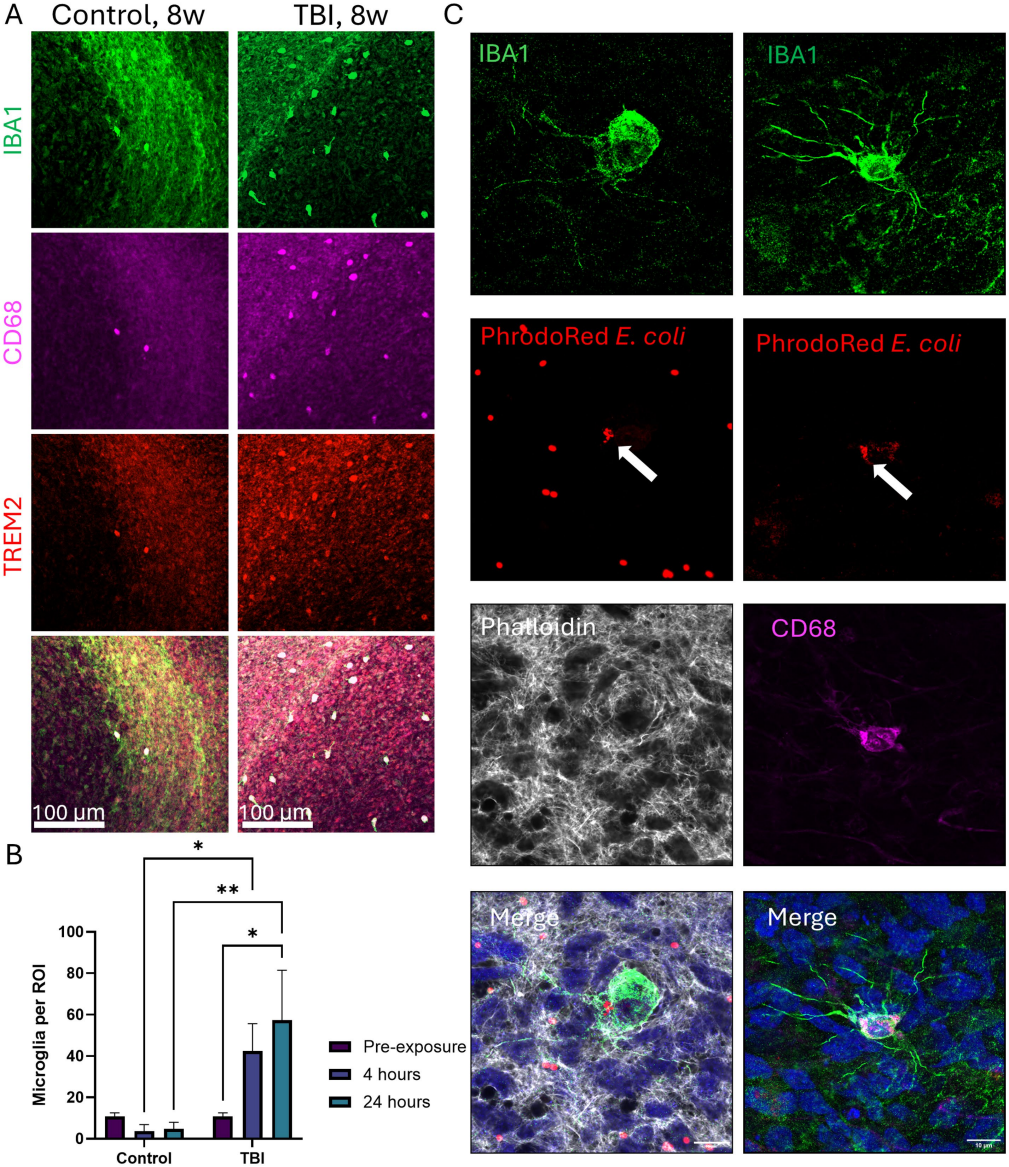
Microglia behavior in the setting of traumatic brain injury (TBI) and their phagocytosis ability. **(A)** Representative images of 8-week μbMPS^96-6^ challenged with TBI vs. control cultures. Cultures were stained with microglia-specific IBA1 (green), TREM2 (red), and CD68 (magenta). The whole surface of each μbMPS was imaged (10X) on an Olympus FVS3000R, and a blinded observer counted microglia on the visible surface. The scale bar is 100 μm **(B)**. Quantification of microglia counts per region of interest on the organoid surface with and without TBI challenge. Data represent the number of microglia on the surface from two independent experiments, with eight μbMPS per condition in total. Statistical significance was evaluated with a two-way ANOVA with *post hoc* Šídák’s multiple comparisons test to time 0 (pre-challenge) and between conditions (+/− TBI) at matched time points. **(C)** Z-stack images obtained from three-week-old μbMPS^96-6^ exposed to PHrodo red *E. coli* bioparticles (red). PHrodo red is colocalized with IBA1 (green) and CD68 (magenta). Cellular filaments are visualized with phalloidin (grey). The IBA1 channel was processed with a 3-pixel median filter to reduce antibody noise. The scale bar is 10 μm.

To confirm that microglia in μbMPS^96-6^ can respond to their environment, we challenged them with a Traumatic Brain Injury (TBI) model. The TBI protocol was modified from a previously published protocol (Ortega-Villa et al., 2021), where it is characterized as a sustained compression injury of moderate severity. Here, the absence of surrounding hydrogel results in a larger force; therefore, we consider this a severe compression injury. A total of 32 μbMPS^96-6^ were randomly selected from the same pool; 16 were challenged with TBI via centrifugation, while the other 16 were not centrifuged. Samples were collected before any manipulation and after 4 and 24 h post-centrifugation. Although no significant changes in microglia morphology were observed, TBI-treated μbMPS^96-6^ exhibited a statistically significant accumulation of microglia on the surface of the organoid z-stack, indicating that microglia traversed to the outer layers of the bMPS to respond to surface neuronal injury (Figures 9A,B).

Next, to verify whether microglia in μbMPS^96-6^ are capable of phagocytosis, we exposed the cultures to 1 mg/mL pHrodo Red *E. coli* particles for 3 h. Z-stacks were obtained of the whole cell (including projections) to confirm that the bioparticles were internalized within the 3D structure of the cell body, rather than in front of or behind the cell. Bioparticles within the microglia appear smaller than surrounding particles, likely representing partial digestion, and colocalize with CD68 labeling of the lysosome or endosome (Figure 9C). While we would expect *E. coli* bioparticles to be phagocytosed rather than internalized during endocytosis, CD68 also labels endosomes. To ensure that pHrodo Red bioparticles are truly internalized into lysosomal compartments, it may be beneficial to label cells with Lysotracker or lysosome-specific proteins such as LAMP1/2.

**Figure 10:**
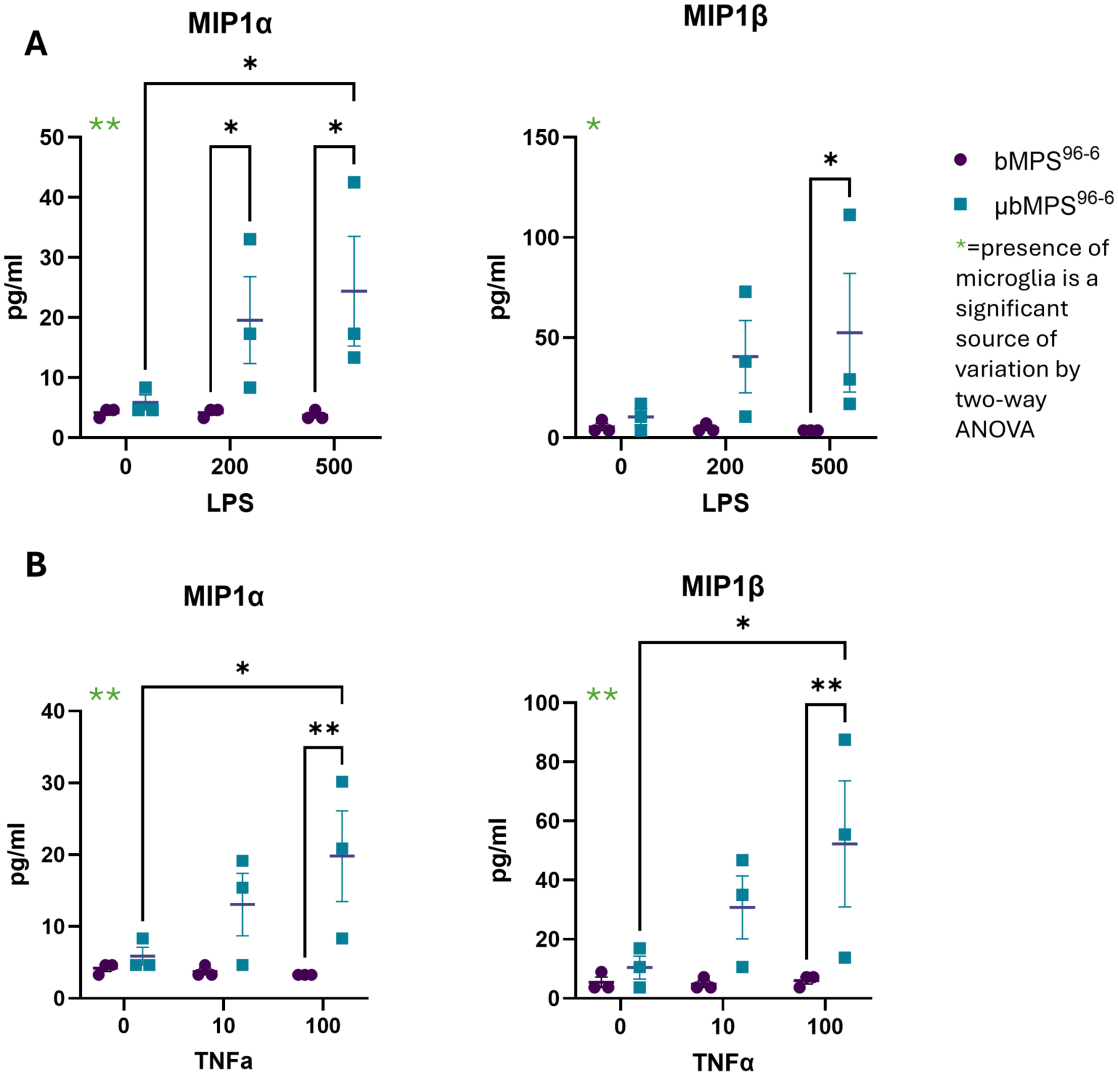
Microglia-specific cytokine release in response to **(A)** LPS (0, 200, and 500 ng/mL) and **(B)** TNFα (0, 10, and 100 ng/mL). MIP1a and MIP1b levels were quantified in three biological replicates per condition. Statistical significance was determined using two-way ANOVA followed by Tukey’s post-hoc test, * *p* < 0.05, ** *p* < 0.01.

Finally, we measured secreted cytokines and chemokines (Supplementary Table S3) in 8-week-old bMPS^96-6^ and μbMPS^96-6^ following exposure to lipopolysaccharide, a component of the cell wall of *E. coli* bacteria (Figure 10A), and TNFα cytokine (Figure 10B). We identified a statistically significant increase in two microglia-specific cytokines, MIP-1α and MIP-1β, only in μbMPS^96-6^; the cytokine flux was absent in non-microglia-containing bMPS (bMPS^96-6^). Low concentrations measured can reflect the low number of microglia (2.5%) per bMPS and low number of bMPS in a large volume of media (2 mL) to allow for standard maintenance on gyratory shaking in a 6-well plate during exposure. In addition, presence of trace endotoxin in bMPS differentiation medium can attenuate the response to LPS challenges. Subsequent studies will control and eliminate endotoxin from differentiation media to mitigate this effect.

## Discussion

Microglia are intricately involved in many aspects of brain development, neuroinflammation, and mechanisms of neurotoxicity, highlighting the importance of their presence in neural models. During human brain development, microglia progenitors arise from the yolk sac in the early days of embryogenesis and migrate into the embryonic brain by week 4.5, where they further proliferate and mature (Monier et al., 2007). Considering their developmental origin, they will naturally be absent in standard neuro-ectodermal lineage-derived models and need to be integrated separately.

Many other groups have developed a variety of microglia-containing brain organoids (Fagiani et al., 2024; Wenzel et al., 2024; Ormel et al., 2018; Bodnar et al., 2021; Popova et al., 2021; Park et al., 2023; Sabate-Soler et al., 2022; Schafer et al., 2023; Buonfiglioli et al., 2025; Fagerlund et al., 2021; Cakir et al., 2022; Kalpana et al., 2025; Wu et al., 2024; Farahani et al., 2023; Xu et al., 2021; Song et al., 2019; Abreu et al., 2018); however, we demonstrated that in our model, the environment provided by the other neural cells is sufficient for microglia maturation and long-term survival without adjusting neural or bMPS differentiation media. This is a prominent strength of our model: reducing the cost of media while also suggesting that the neural cells are producing cytokines necessary for microglia survival, as they do in the developing human brain. We have presented here a model with a standardized tunable ratio of cells and demonstrated that microglia mature within the bMPS, survive long-term (at least 9 weeks post-integration), and display appropriate functioning and typical morphologies, with no addition of microglia-specific supplements (growth factors, cytokines, and chemokines) or changes to the differentiation medium.

We used 96-well U-bottom plates to aggregate neural progenitors and premature microglia. This approach allowed us to customize the ratio of these two cell types for specific applications and to have precise control over the size of the resulting bMPS, which are highly standardized and can be modified as needed, such as to accommodate different cell growth rates of different donor iPSCs. Additionally, this model enables users to bank frozen aliquots of both NPCs and PMs, allowing for rapid generation of co-cultures, which can save at least 4–5 weeks per experiment compared to directly differentiating from iPSCs.

Furthermore, by controlling the number of microglia per bMPS, we can evaluate microglia migration to the core or surface of the bMPS, as the total number of cells per organoid is highly reproducible between different MPS. Additionally, integrating separate cultures of PMs and neuronal progenitors facilitates the use of live-labeling strategies for cell tracking or time-course image analysis (Supplementary Figure S3B and Supplementary Video 1). Microglia are known to proliferate at a very slow rate, with a turnover in the adult human brain of approximately 28% per year, and the average microglia persists in the brain for over 4 years (Réu et al., 2017). With this in mind, the number of microglia per bMPS is likely relatively stable, beginning at 1000 cells per μbMPS^96-6^ at the day of integration. In contrast, other cell types proliferate significantly in culture, leading to, over time, a lower relative number of microglia cells. This is supported by the lower number of microglia visible in the μbMPS^96-6^ at mature time points compared to earlier time points. The microglia are distributed across a growing system, but, as shown in the model for traumatic brain injury (Figure 9), they are present, likely towards the core of the bMPS, and are able to respond to injuries. Future studies will focus on characterizing the distribution and morphologies of microglia over time using live cell labeling.

Overall, our model recapitulates the cellular composition observed *in vivo,* with an approximate 1:1 ratio of neurons to non-neuronal cells (Azevedo et al., 2009). *In vivo,* glial proportions range from 72 to 19%, depending on the brain region (Tan et al., 2020). Microglia comprise an average of 2.5% at 6 weeks in our model, which falls within the *in vivo* range of 0.5–15% (Frost and Schafer, 2016; Mittelbronn et al., 2001). Earlier timepoints can be used if higher microglial densities are of interest.

Other models for microglia incorporation into bMPS have been published and have been compared in Table 1 and Supplementary Figure S1. These models span a variety of brain regions, include both undirected and directed differentiations, include microglia from a variety of homemade and commercial sources, and include microglia for as short as 24 h or throughout the entire culture period. Each model may be differentially suited for a different research questions. Multiple groups (Wenzel et al., 2024; Ormel et al., 2018; Bodnar et al., 2021) have demonstrated spontaneous differentiation of microglia cells within neural organoids during neuro-ectoderm differentiation from embryoid bodies. The presence of innately differentiating microglia cells is a strength of these methods. Still, standardization can be challenging, as the presence of microglia during ectodermal lineage differentiation is not fully understood and potentially can arise from the crossover of the cells from mesodermal lineage within the embryoid bodies, which can also contribute to inter-organoid variability, particularly across different cell lines.

Several protocols have addressed this by adding microglia to already formed brain organoids (Fagiani et al., 2024; Wenzel et al., 2024; Popova et al., 2021; Park et al., 2023; Sabate-Soler et al., 2022; Schafer et al., 2023; Buonfiglioli et al., 2025; Fagerlund et al., 2021; Kalpana et al., 2025; Wu et al., 2024; Farahani et al., 2023; Abreu et al., 2018), which allows microglia to be applied to a variety of different organoid and assembloid generation protocols. However, it can result in a variable number of microglia per organoid or asymmetric distribution between organoids. Additionally, including microglia at later stages, for only a portion of the time course, does not allow for the study of microglial roles in the early events of neural differentiation. Thus, these models may be more suitable for studying the role of microglia in response to a specific exposure as a neuroinflammation mediator, rather than the role of microglia in neurodevelopment. Some protocols include microglia during organoid formation (Wenzel et al., 2024; Cakir et al., 2022; Kalpana et al., 2025; Xu et al., 2021) with variation in survival of microglia or required additional growth factors.

Almost all protocols, regardless of integration strategy, introduce external microglia-specific factors into the medium during the integration. Most commonly, these are microglia-specific cytokines (Park et al., 2023; Sabate-Soler et al., 2022; Schafer et al., 2023; Buonfiglioli et al., 2025; Kalpana et al., 2025; Wu et al., 2024; Farahani et al., 2023; Muffat et al., 2016; Xu et al., 2021) but also include lowered heparin (Ormel et al., 2018; Bodnar et al., 2021) or doxycycline induction of CRISPR-integrated genes to drive microglia differentiation (Cakir et al., 2022) (Table 1). Some added factors have been found to impact the differentiation trajectories of other cell types, such as the dopaminergic system (Sabate-Soler et al., 2022), and must be carefully controlled for. Gene editing, with CRISPR or other tools such as TALENs, to produce cell lines with an inducible differentiation to microglia fate, is an enticing strategy to facilitate the rapid production of microglia-like cells (Cakir et al., 2022; Dräger et al., 2022). However, these methods will be challenging to implement across multiple cell lines due to the inherent difficulty of gene editing and also carry the potential for untargeted effects (Walters et al., 2016).

The primary goal of our study was to develop a method for high-throughput testing of immunocompetent brain microphysiological systems derived from iPSCs that can easily be adapted to different cell lines for testing neurotoxic compounds and neurodevelopmental disorders. Our model needed to be easily adaptable, high-throughput, avoid potentially costly additions to media, and exhibit survival of microglia throughout the entire culture period.

In our model, we modified the protocol described by Xu et al. (2021). We showed that microglia matured in the milieu of the μbMPS and survive for at least 9 weeks post-integration of NPC and PMs, which is sufficient time to develop robust spontaneous electrical activity, as well as differentiation of mature cell types: glutamatergic and GABAergic neurons, astrocytes, and oligodendrocytes (Romero et al., 2023; Alam El Din et al., 2025). We have demonstrated that the microglia within the μbMPS^96-6^ express markers specific to mature microglia, can respond to external factors, are capable of phagocytosis of *E. coli* particles, and can move throughout the μbMPS^96-6^ to react to injuries.

Notably, in the phagocytosis assay, some particles were visible outside of microglia and may have been internalized by other cell types. While microglia are known to be responsible for the bulk of phagocytosis in the brain, of both synapses and pathogenic particles, other cell types, e.g., astrocytes, have been found to participate in phagocytosis (Crespo-Castrillo et al., 2020). We have observed both ramified and ameboid microglia morphologies at different stages of differentiation. Apoptosis is a natural process during neuronal development and is expected during the prolonged culture of bMPS, which is associated with a certain percentage of cell death. The presence of ameboid microglia suggests that they have been activated by the potential traces of endotoxin in cell culture medium and/or internal factors, which may include apoptosis, mild hypoxia as the bMPS grow larger, or shear stress generated by the gyratory shaking. Future work will pursue further characterization of microglia within the bMPS beyond basic activity, including responses to exposures, single-cell transcriptomic analysis of the μbMPSs, as well as a broad panel of cytokine/chemokine stimuli response profiles.

Additionally, while we have demonstrated the expression of mature and specific markers of microglia through immunofluorescence, flow cytometry of microglia matured within the bMPS would be a stronger characterization strategy. This would encompass both the abundance of cell types as well as potential diversity in these populations. However, our current methods for bMPS dissociation are ineffective for the substantially larger bMPS^96-6^, which leads to poor cell viability and would affect lineage distribution measurements. We are pursuing further optimization of flow cytometry for the bMPS^96-6^.

Microglia play many distinct roles throughout development, and early-life stressors may disrupt their maturation. As this model includes microglia throughout the entire period of bMPS culture, it is highly relevant for studying neurodevelopmental disorders, in which microglia may contribute to pathogenesis at different developmental windows and stages of neurogenesis. It is also valuable for neurotoxicology research, as microglia may respond to a stressor at one time point in ways that shape their later behavior, contributing to toxicological and phenotypical effects. In addition, this model offers higher throughput analysis and may aid in the identification of poorly studied compounds with potential adverse developmental neurotoxic effects (Kincaid et al., 2023).

## Conclusion

Taken together, our results demonstrate that the neural milieu alone is sufficient for microglia to mature, survive, and function, with no additional microglia-specific factors needed when co-cultured with brain MPSs. Moreover, our protocol is standardized, faster than other differentiation methods, scalable, and adaptable across different cell lines.

## Data Availability

The data discussed in this publication have been deposited in NCBI’s Gene Expression Omnibus and are accessible through GEO Series accession number GSE307124 (https://www.ncbi.nlm.nih.gov/geo/query/acc.cgi?acc=GSE307124).

## References

[ref1] Abreu C. M. Gama L. Krasemann S. Chesnut M. Odwin-Dacosta S. Hogberg H. T. . (2018). Microglia Increase Inflammatory Responses in iPSC-Derived Human BrainSpheres. Front. Microbiol. 9:9. doi: 10.3389/fmicb.2018.02766, PMID: 30619100 PMC6296317

[ref2] Alam El Din D. M. Moenkemoeller L. Loeffler A. Habibollahi F. Schenkman J. Mitra A. . (2025). Human neural organoid microphysiological systems show the building blocks necessary for basic learning and memory. Commun Biol. 8:1237. doi: 10.1038/s42003-025-08632-5 40819006 PMC12357958

[ref3] Arnoux I. Audinat E. (2015). Fractalkine Signaling and Microglia Functions in the Developing Brain. Neural Plast. 2015, 1–8. doi: 10.1155/2015/689404, PMID: 26347402 PMC4539507

[ref4] Azevedo F. A. C. Carvalho L. R. B. Grinberg L. T. Farfel J. M. Ferretti R. E. L. Leite R. E. P. . (2009). Equal numbers of neuronal and nonneuronal cells make the human brain an isometrically scaled-up primate brain. J. Comp. Neurol. 513, 532–541. doi: 10.1002/cne.21974, PMID: 19226510

[ref5] Bardou P. Mariette J. Escudié F. Djemiel C. Klopp C. (2014). jvenn: an interactive Venn diagram viewer. BMC Bioinformatics 15:293. doi: 10.1186/1471-2105-15-293, PMID: 25176396 PMC4261873

[ref6] Bodnar B. Zhang Y. Liu J. Lin Y. Wang P. Wei Z. . (2021). Novel Scalable and Simplified System to Generate Microglia-Containing Cerebral Organoids From Human Induced Pluripotent Stem Cells. Front. Cell. Neurosci. 15:15. doi: 10.3389/fncel.2021.682272, PMID: 34290591 PMC8288463

[ref8] Buonfiglioli A. Kübler R. Missall R. De Jong R. Chan S. Haage V. . (2025). A microglia-containing cerebral organoid model to study early life immune challenges. Brain Behav. Immun. 123, 1127–1146. doi: 10.1016/j.bbi.2024.11.008, PMID: 39500415 PMC11753195

[ref9] Cakir B. Tanaka Y. Kiral F. R. Xiang Y. Dagliyan O. Wang J. . (2022). Expression of the transcription factor PU.1 induces the generation of microglia-like cells in human cortical organoids. Nat. Commun. 13:430. doi: 10.1038/s41467-022-28043-y, PMID: 35058453 PMC8776770

[ref10] Computational Genomics Research. (2019). The Smith lab. Available online at: https://smithlabresearch.org/software/preseq/ (Accessed May 01, 2025).

[ref11] Crespo-Castrillo A. Garcia-Segura L. M. Arevalo M. A. (2020). The synthetic steroid tibolone exerts sex-specific regulation of astrocyte phagocytosis under basal conditions and after an inflammatory challenge. J. Neuroinflammation 17:37. doi: 10.1186/s12974-020-1719-6, PMID: 31992325 PMC6986022

[ref12] Dobin A. Davis C. A. Schlesinger F. Drenkow J. Zaleski C. Jha S. . (2013). STAR: ultrafast universal RNA-seq aligner. Bioinformatics 29, 15–21. doi: 10.1093/bioinformatics/bts635, PMID: 23104886 PMC3530905

[ref13] Dräger N. M. Sattler S. M. Huang C. T. L. Teter O. M. Leng K. Hashemi S. H. . (2022). A CRISPRi/a platform in human iPSC-derived microglia uncovers regulators of disease states. Nat. Neurosci. 25, 1149–1162. doi: 10.1038/s41593-022-01131-4, PMID: 35953545 PMC9448678

[ref14] Ewels P. A. Peltzer A. Fillinger S. Patel H. Alneberg J. Wilm A. . (2020). The nf-core framework for community-curated bioinformatics pipelines. Nat. Biotechnol. 38, 276–278. doi: 10.1038/s41587-020-0439-x, PMID: 32055031

[ref15] Fagerlund I. Dougalis A. Shakirzyanova A. Gómez-Budia M. Pelkonen A. Konttinen H. . (2021). Microglia-like Cells Promote Neuronal Functions in Cerebral Organoids. Cells 11:124. doi: 10.3390/cells11010124, PMID: 35011686 PMC8750120

[ref16] Fagiani F. Pedrini E. Taverna S. Brambilla E. Murtaj V. Podini P. . (2024). A glia-enriched stem cell 3D model of the human brain mimics the glial-immune neurodegenerative phenotypes of multiple sclerosis. Cell Rep. Med. 5:101680. doi: 10.1016/j.xcrm.2024.101680, PMID: 39121861 PMC11384947

[ref17] Farahani N. I. Chan J. Añonuevo A. Chew L. H. Knock E. (2023). “Generating Human Pluripotent Stem Cell-Derived Neural AssemBloids™ to Model Interneuron Migration and Immune Cell Interactions” in Emerging model organisms, Eds. Wang W. Rohner N. Neuromethods Wang Y., 194, 307–324. New York, NY: Humana. doi: 10.1007/978-1-0716-2875-1_21

[ref18] Frost J. L. Schafer D. P. (2016). Microglia: Architects of the Developing Nervous System. Trends Cell Biol. 26, 587–597. doi: 10.1016/j.tcb.2016.02.006, PMID: 27004698 PMC4961529

[ref19] García-Alcalde F. Okonechnikov K. Carbonell J. Cruz L. M. Götz S. Tarazona S. . (2012). Qualimap: evaluating next-generation sequencing alignment data. Bioinformatics 28, 2678–2679. doi: 10.1093/bioinformatics/bts503, PMID: 22914218

[ref20] Ginhoux F. Lim S. Hoeffel G. Low D. Huber T. (2013). Origin and differentiation of microglia. Front. Cell. Neurosci. 7:7. doi: 10.3389/fncel.2013.00045, PMID: 23616747 PMC3627983

[ref21] González-Cruz R. D. Wan Y. Burgess A. Calvao D. Renken W. Vecchio F. . (2024). Cortical spheroids show strain-dependent cell viability loss and neurite disruption following sustained compression injury. PLoS One 19:e0295086. doi: 10.1371/journal.pone.0295086, PMID: 39159236 PMC11332998

[ref22] Honda T. Inagawa H. (2023). Utility of In Vitro Cellular Models of Low-Dose Lipopolysaccharide in Elucidating the Mechanisms of Anti-Inflammatory and Wound-Healing-Promoting Effects of Lipopolysaccharide Administration In Vivo. Int. J. Mol. Sci. 24:14387. doi: 10.3390/ijms241814387, PMID: 37762690 PMC10532185

[ref23] Kalpana K. Rao C. Semrau S. Zhang B. Noggle S. Fossati V. (2025). Generating Neuroimmune Assembloids Using Human Induced Pluripotent Stem Cell (iPSC)-Derived Cortical Organoids and Microglia. Methods Mol Biol. 2951, 139–158. doi: 10.1007/7651_2024_554 38976205

[ref24] Kincaid B. Piechota P. Golden E. Maertens M. Hartung T. Maertens A. (2023). Using in silico tools to predict flame retardant metabolites for more informative exposomics-based approaches. Front. Toxicol. 5:5. doi: 10.3389/ftox.2023.1216802, PMID: 37908592 PMC10613991

[ref25] Krueger F. James F. Ewels P. Afyounian E. Weinstein M. Schuster-Boeckler B. . Trim Galore. (2023). Babraham Institute, Cambridge England.

[ref26] Li H. Handsaker B. Wysoker A. Fennell T. Ruan J. Homer N. . (2009). The Sequence Alignment/Map format and SAMtools. Bioinformatics 25, 2078–2079. doi: 10.1093/bioinformatics/btp352, PMID: 19505943 PMC2723002

[ref27] Luo Y. Wang Z. (2024). The Impact of Microglia on Neurodevelopment and Brain Function in Autism. Biomedicine 12:210. doi: 10.3390/biomedicines12010210, PMID: 38255315 PMC10813633

[ref28] Mangiola S. Molania R. Dong R. Doyle M. A. Papenfuss A. T. (2021). tidybulk: an R tidy framework for modular transcriptomic data analysis. Genome Biol. 22:42. doi: 10.1186/s13059-020-02233-7, PMID: 33482892 PMC7821481

[ref29] Martin F. J. Amode M. R. Aneja A. Austine-Orimoloye O. Azov A. G. Barnes I. . (2023). Ensembl 2023. Nucleic Acids Res. 51, D933–D941. doi: 10.1093/nar/gkac958, PMID: 36318249 PMC9825606

[ref30] Martinez A. Hériché J. K. Calvo M. Tischer C. Otxoa-de-Amezaga A. Pedragosa J. . (2023). Characterization of microglia behaviour in healthy and pathological conditions with image analysis tools. Open Biol. 13:220200. doi: 10.1098/rsob.220200, PMID: 36629019 PMC9832574

[ref31] Matejuk A. Ransohoff R. M. (2020). Crosstalk between astrocytes and microglia: an overview. Front. Immunol. 11:1416. doi: 10.3389/fimmu.2020.01416 32765501 PMC7378357

[ref32] Miller-Rhodes P. (2022). A guide to microglia markers. San Francisco: Life Science Articles.

[ref33] Mittelbronn M. Dietz K. Schluesener H. J. Meyermann R. (2001). Local distribution of microglia in the normal adult human central nervous system differs by up to one order of magnitude. Acta Neuropathol. 101, 249–255. doi: 10.1007/s004010000284, PMID: 11307625

[ref34] Monier A. Adle-Biassette H. Delezoide A. L. Evrard P. Gressens P. Verney C. (2007). Entry and Distribution of Microglial Cells in Human Embryonic and Fetal Cerebral Cortex. J. Neuropathol. Exp. Neurol. 66, 372–382. doi: 10.1097/nen.0b013e3180517b46, PMID: 17483694

[ref35] Morales Pantoja I. E. Ding L. Leite P. E. C. Marques S. A. Romero J. C. Alam El Din D. . (2024). A Novel Approach to Increase Glial Cell Populations in Brain Microphysiological Systems. Adv. Biol. 8:e2300198. doi: 10.1002/adbi.202300198, PMID: 38062868 PMC11156795

[ref36] Mordelt A. de Witte L. D. (2023). Microglia-mediated synaptic pruning as a key deficit in neurodevelopmental disorders: Hype or hope? Curr. Opin. Neurobiol. 79:102674. doi: 10.1016/j.conb.2022.102674 36657237

[ref37] Moreno Manrique J. F. Voit P. R. Windsor K. E. Karla A. R. Rodriguez S. R. Beaudoin G. M. J. (2021). SynapseJ: An Automated, Synapse Identification Macro for ImageJ. Front. Neural Circuits 15:15. doi: 10.3389/fncir.2021.731333, PMID: 34675779 PMC8524137

[ref38] Muffat J. Li Y. Yuan B. Mitalipova M. Omer A. Corcoran S. . (2016). Efficient derivation of microglia-like cells from human pluripotent stem cells. Nat. Med. 22, 1358–1367. doi: 10.1038/nm.4189, PMID: 27668937 PMC5101156

[ref39] Ormel P. R. Vieira de Sá R. van Bodegraven E. J. Karst H. Harschnitz O. Sneeboer M. A. M. . (2018). Microglia innately develop within cerebral organoids. Nat. Commun. 9:4167. doi: 10.1038/s41467-018-06684-2, PMID: 30301888 PMC6177485

[ref40] Ortega-Villa A. M. Liu D. Ward M. H. Albert P. S. (2021). New insights into modeling exposure measurements below the limit of detection. Environ. Epidemiol. 5:e116. doi: 10.1097/EE9.0000000000000116, PMID: 33778356 PMC7939440

[ref41] Palpagama T. H. Waldvogel H. J. Faull R. L. M. Kwakowsky A. (2019). The Role of Microglia and Astrocytes in Huntington’s Disease. Front. Mol. Neurosci. 12:12. doi: 10.3389/fnmol.2019.00258, PMID: 31708741 PMC6824292

[ref42] Park D. S. Kozaki T. Tiwari S. K. Moreira M. Khalilnezhad A. Torta F. . (2023). iPS-cell-derived microglia promote brain organoid maturation via cholesterol transfer. Nature 623, 397–405. doi: 10.1038/s41586-023-06713-1, PMID: 37914940

[ref43] Pașca S. P. Arlotta P. Bateup H. S. Camp J. G. Cappello S. Gage F. H. . (2022). A nomenclature consensus for nervous system organoids and assembloids. Nature 609, 907–910. doi: 10.1038/s41586-022-05219-6, PMID: 36171373 PMC10571504

[ref44] Patro R. Duggal G. Love M. I. Irizarry R. A. Kingsford C. (2017). Salmon provides fast and bias-aware quantification of transcript expression. Nat. Methods 14, 417–419. doi: 10.1038/nmeth.4197, PMID: 28263959 PMC5600148

[ref7] Picard Toolkit. (2019). Broad Institute. Picard. Available online at: https://broadinstitute.github.io/picard (Accessed May 01, 2025).

[ref45] Popova G. Soliman S. S. Kim C. N. Keefe M. G. Hennick K. M. Jain S. . (2021). Human microglia states are conserved across experimental models and regulate neural stem cell responses in chimeric organoids. Cell Stem Cell 28, 2153–2166.e6. doi: 10.1016/j.stem.2021.08.015, PMID: 34536354 PMC8642295

[ref46] Quinlan A. R. Hall I. M. (2010). BEDTools: a flexible suite of utilities for comparing genomic features. Bioinformatics 26, 841–842. doi: 10.1093/bioinformatics/btq033, PMID: 20110278 PMC2832824

[ref47] Réu P. Khosravi A. Bernard S. Mold J. E. Salehpour M. Alkass K. . (2017). The Lifespan and Turnover of Microglia in the Human Brain. Cell Rep. 20, 779–784. doi: 10.1016/j.celrep.2017.07.004, PMID: 28746864 PMC5540680

[ref48] Romero J. C. Berlinicke C. Chow S. Duan Y. Wang Y. Chamling X. . (2023). Oligodendrogenesis and myelination tracing in a CRISPR/Cas9-engineered brain microphysiological system. Front. Cell. Neurosci. 16:1094291. doi: 10.3389/fncel.2022.1094291, PMID: 36744062 PMC9893511

[ref49] Sabate-Soler S. Nickels S. L. Saraiva C. Berger E. Dubonyte U. Barmpa K. . (2022). Microglia integration into human midbrain organoids leads to increased neuronal maturation and functionality. Glia 70, 1267–1288. doi: 10.1002/glia.24167, PMID: 35262217 PMC9314680

[ref50] Sayols S. Scherzinger D. Klein H. (2016). dupRadar: a Bioconductor package for the assessment of PCR artifacts in RNA-Seq data. BMC Bioinformatics 17:428. doi: 10.1186/s12859-016-1276-2, PMID: 27769170 PMC5073875

[ref51] Schafer D. P. Lehrman E. K. Stevens B. (2013). The “quad‐partite” synapse: Microglia‐synapse interactions in the developing and mature CNS. Glia 61, 24–36. doi: 10.1002/glia.22389, PMID: 22829357 PMC4082974

[ref52] Schafer S. T. Mansour A. A. Schlachetzki J. C. M. Pena M. Ghassemzadeh S. Mitchell L. . (2023). An in vivo neuroimmune organoid model to study human microglia phenotypes. Cell 186, 2111–2126.e20. doi: 10.1016/j.cell.2023.04.022, PMID: 37172564 PMC10284271

[ref53] Schmittgen T. D. Livak K. J. (2001). Analysis of relative gene expression data using real-time quantitative PCR and the 2(-Delta Delta C(T)) Method. Methods 25, 383–385. doi: 10.1006/meth.2001.1262, PMID: 11846609

[ref54] Song L. Yuan X. Jones Z. Vied C. Miao Y. Marzano M. . (2019). Functionalization of Brain Region-specific Spheroids with Isogenic Microglia-like Cells. Sci. Rep. 9:11055. doi: 10.1038/s41598-019-47444-6, PMID: 31363137 PMC6667451

[ref55] Tan Y. L. Yuan Y. Tian L. (2020). Microglial regional heterogeneity and its role in the brain. Mol. Psychiatry 25, 351–367. doi: 10.1038/s41380-019-0609-8, PMID: 31772305 PMC6974435

[ref56] Trainor A. R. MacDonald D. S. Penney J. (2024). Microglia: roles and genetic risk in Parkinson’s disease. Front. Neurosci. 18:18. doi: 10.3389/fnins.2024.1506358, PMID: 39554849 PMC11564156

[ref57] Vasilescu C. Olteanu M. Flondor P. (2009). How relevant are in vivo and in vitro studies for clinical sepsis A mathematical model of LPS signaling based on endotoxin tolerance. Chirurgia: Official journal of the romanian society of surgery, 2. 19499663

[ref58] Walters B. J. Azam A. B. Gillon C. J. Josselyn S. A. Zovkic I. B. (2016). Advanced In vivo Use of CRISPR/Cas9 and Anti-sense DNA Inhibition for Gene Manipulation in the Brain. Front. Genet. 6:362. doi: 10.3389/fgene.2015.00362 26793235 PMC4709581

[ref60] Wang L. Wang S. Li W. (2012). RSeQC: quality control of RNA-seq experiments. Bioinformatics 28, 2184–2185. doi: 10.1093/bioinformatics/bts356, PMID: 22743226

[ref61] Wenzel T. J. Desjarlais J. D. Mousseau D. D. (2024). Human brain organoids containing microglia that have arisen innately adapt to a β-amyloid challenge better than those in which microglia are integrated by co-culture. Stem Cell Res Ther 15:258. doi: 10.1186/s13287-024-03876-0, PMID: 39135132 PMC11320858

[ref62] Wickstead E. S. (2023). Using Stems to Bear Fruit: Deciphering the Role of Alzheimer’s Disease Risk Loci in Human-Induced Pluripotent Stem Cell-Derived Microglia. Biomedicine 11:2240. doi: 10.3390/biomedicines11082240, PMID: 37626736 PMC10452566

[ref63] Wu J. Zhang J. Chen X. Wettschurack K. Que Z. Deming B. A. . (2024). Microglial over-pruning of synapses during development in autism-associated SCN2A-deficient mice and human cerebral organoids. Mol. Psychiatry 29, 2424–2437. doi: 10.1038/s41380-024-02518-4, PMID: 38499656

[ref64] Xu R. Boreland A. J. Li X. Erickson C. Jin M. Atkins C. . (2021). Developing human pluripotent stem cell-based cerebral organoids with a controllable microglia ratio for modeling brain development and pathology. Stem Cell Reports 16, 1923–1937. doi: 10.1016/j.stemcr.2021.06.011, PMID: 34297942 PMC8365109

[ref65] Yücel G. Zhao Z. El-Battrawy I. Lan H. Lang S. Li X. . (2017). Lipopolysaccharides induced inflammatory responses and electrophysiological dysfunctions in human-induced pluripotent stem cell derived cardiomyocytes. Sci. Rep. 7:2935. doi: 10.1038/s41598-017-03147-4, PMID: 28592841 PMC5462745

