# Supplementary material for: Microglia-containing neural organoids as brain microphysiological systems for long-term culture
Source: Front Cell Neurosci. 2025 Oct 2;19:1616470. doi: 10.3389/fncel.2025.1616470 (PMC12528120; doi:10.3389/fncel.2025.1616470)
Supplement: Supplementary file 1 [file Data_Sheet_1.docx]

**Microglia-containing neural organoids as brain Microphysiologic Systems for long-term culture**

## Alex Rittenhouse^1^, Caroline Krall^1, 2^, Jesse Plotkin^1^, Dowlette-Mary Alam El Din^1^, Breanne Kincaid^1^, Jason Laird^1^, and Lena Smirnova^1^

##

## ^1^ Center for Alternatives to Animal Testing, Environmental Health and Engineering Department, Bloomberg School of Public Health, Johns Hopkins University

## ^2^ Department of Molecular and Comparative Pathobiology, Johns Hopkins University School of Medicine, 733 N. Broadway, Baltimore, MD, 21205, USA

**Supplementary material**

**Supplementary Table S1.** TaqMan gene expression assays for Real-Time PCR

| **Gene** | **Catalog** | **Cell Target** |
| --- | --- | --- |
| Myelin Basic Protein (*MBP*) | Hs00921945 | Oligodendrocytes |
| Glial Fibrillary Acidic Protein (*GFAP*) | Hs00909233 | Astrocytes |
| Microtubule Associated Protein (*MAP2*) | Hs00258900 | Neurons |
| Synaptophysin (*SYP*) | Hs00300531 | Neurons |
| Actin Beta (*ACTB*) | Hs01060665 | Total cells |
| *18S* | Hs99999901 | Total Cells |

**Supplementary Table S2.** List of primary and secondary Antibodies

| **Antibody** | **Host** | **Product** | **Concentration** |
| --- | --- | --- | --- |
| PRIMARY | | | |
| IBA1 | Chicken | Aves #02200 | 1:400 |
| NF200 | Rabbit | Sigma #N4142 | 1:250 |
| GFAP | Rabbit | DAKO #Z0334 | 1:200 |
| β-III-Tubulin | Mouse | Sigma #T7056 | 1:1500 |
| TREM2 | Rat | R&D #MAB17291 | 1:75 |
| Synapsin 1 | Rabbit | EMD Millipore #AB1543P | 1:400 |
| Homer 1 | Mouse | Synaptic Systems #160-011 | 1:100 |
| CD68 | Mouse | Dako | 1:75 |
| P2Rγ12 | Rabbit | Invitrogen | 1:500 |
| O4 | Mouse | R&D #MAB1326 | 1:200 |
| Secondary | | | |
| Anti-chicken 647 | Goat | Invitrogen | 1:500 |
| Anti-rat 488 | Goat | Invitrogen | 1:500 |
| Anti-rabbit 568 | Goat | Invitrogen | 1:500 |
| Anti-mouse 568 | Goat | Invitrogen | 1:500 |
| Anti-rabbit 488 | Goat | Invitrogen | 1:500 |
| Anti-mouse 488 | Goat | Invitrogen | 1:500 |
| Anti-mouse 405 | Goat | Invitrogen | 1:500 |
| Anti-chicken 488 | Goat | Invitrogen | 1:500 |
| COUNTERSTAIN | | | |
| Hoechst33342 | | ThermoFisher Scientific #33342 | 1:10,000 |
| Conjugated antibodies and fluorescent markers | | | |
| FITC anti-CD43 | | BD Biosciences 555475 | |
| APC anti-CD45 | | BD Biosciences 560973 | |
| PE anti-CD34 | | BD Biosciences 560941 | |
| CD11b | | BD Biosciences 561015 | |
| Phalloidin 647 | | ThermoFisher Scientific #A22287 | |

**Supplementary Table S3.** List of evaluated cytokines, chemokines and growth factors**.**

| Cytokines | G-CSF, *GM-CSF, IFN-α, IFN-γ, IL-1β, IL-1RA , IL-2, IL-2R, IL-4, IL-5, IL-6, IL-7, IL-8, IL-10, IL-12 (p40/p70), IL-13, IL-15, IL-17, TNF-α |
| --- | --- |
| Chemokines | Eotaxin, IP-10, MCP-1, MIG, MIP-1α, MIP-1β, RANTES |
| Growth factors: | *EGF, *FGF-basic, *HGF, *VEGF |

*not included in 25-plex

**Supplementary Figure S1.** Visual representation of findings from Supplemental Table 4. Number of papers sharing each of the following categories: media not altered, long-term survival/presence of microglia, microglia present from formation, iPSC-derived microglia. **(B)** Number of papers fulfilling each criterion and **(C)** Number of papers fulfilling only 1, 2, 3, or all criteria.

**Supplementary Figure S2. (A)** Schematic image representing three different integration methods assayed. (1) PMs were added to already formed bMPS in 6-well plates, left static for 24 hours, then returned to gyratory shaking for 4 weeks. (2) NPCs and PMs were added directly to a 6-well dish and placed on gyratory shaking, then cultured for 4 weeks. (3) NPCs and PMs were added to each well of a 96-well dish, left static for 4 days, then pooled into a single well of a 6-well plate and cultured for 4 weeks on gyratory shaking. Figure created in BioRender. Smirnova, L. (2025) https://BioRender.com/cxkatbi. **(B)** Number of processes, outgrowths, or cellular protrusions per bMPS in aggregates cultured in four different plates. **(C)** Diameter (in microns) of individual μbMPS^96-6^ cultured in U-bottom 96-well plates (integration method 3), showing high reproducibility of size in three independent aggregations.

**Supplementary Figure S3. (A)** Cell counts from n=3 individual µbMPS demonstrate an average of 1.9x10^5^ cells per µbMPS^96-6^ with an average of 2.5% microglia. **(B)** Snapshot of the video recording of migrating microglia in a 6-week-old µbMPS^96-6^, recorded over 20 hours at 20X magnification. PMs were labeled prior to integration with NPCs with lipophilic DiD oil (ThermoFisher Scientific, catalog # D307), which allowed to track the microglia over time within the µbMPS^96-6^.
